# Disruption of Toxoplasma gondii-Induced Host Cell DNA Replication Is Dependent on Contact Inhibition and Host Cell Type

**DOI:** 10.1128/msphere.00160-22

**Published:** 2022-05-19

**Authors:** Edwin Pierre-Louis, Menna G. Etheridge, Rodrigo de Paula Baptista, Asis Khan, Nathan M. Chasen, Ronald D. Etheridge

**Affiliations:** a Department of Cellular Biology, Center for Tropical and Emerging Global Diseases (CTEGD), University of Georgia, Athens, Georgia, USA; b Infectious Diseases & Pathology Research Team, Houston Methodist Research Institute, Houston, Texas, USA; c Molecular Parasitology Section, Laboratory of Parasitic Diseases, NIAID, NIH, Bethesda, Maryland, USA; Indiana University School of Medicine

**Keywords:** *Toxoplasma gondii*, cell cycle, cyclin E, FUCCI, S-phase, HCE1/TEEGR, host-parasite interaction, parasite effectors, HCE1, TEEGR

## Abstract

The protozoan Toxoplasma gondii is a highly successful obligate intracellular parasite that, upon invasion of its host cell, releases an array of host-modulating protein effectors to counter host defenses and further its own replication and dissemination. Early studies investigating the impact of T. gondii infection on host cell function revealed that this parasite can force normally quiescent cells to activate their cell cycle program. Prior reports by two independent groups identified the dense granule protein effector HCE1/TEEGR as being solely responsible for driving host cell transcriptional changes through its direct interaction with the cyclin E regulatory complex DP1 and associated transcription factors. Our group independently identified HCE1/TEEGR through the presence of distinct repeated regions found in a number of host nuclear targeted parasite effectors and verified its central role in initiating host cell cycle changes. Additionally, we report here the time-resolved kinetics of host cell cycle transition in response to HCE1/TEEGR, using the fluorescence ubiquitination cell cycle indicator reporter line (FUCCI), and reveal the existence of a block in S-phase progression and host DNA synthesis in several cell lines commonly used in the study of T. gondii. Importantly, we have observed that this S-phase block is not due to additional dense granule effectors but rather is dependent on the host cell line background and contact inhibition status of the host monolayer *in vitro*. This work highlights intriguing differences in the host response to reprogramming by the parasite and raises interesting questions regarding how parasite effectors differentially manipulate the host cell depending on the *in vitro* or *in vivo* context.

**IMPORTANCE** Toxoplasma gondii chronically infects approximately one-third of the global population and can produce severe pathology in immunologically immature or compromised individuals. During infection, this parasite releases numerous host-targeted effector proteins that can dramatically alter the expression of a variety of host genes. A better understanding of parasite effectors and their host targets has the potential to not only provide ways to control infection but also inform us about our own basic biology. One host pathway that has been known to be altered by T. gondii infection is the cell cycle, and prior reports have identified a parasite effector, known as HCE1/TEEGR, as being responsible. In this report, we further our understanding of the kinetics of cell cycle transition induced by this effector and show that the capacity of HCE1/TEEGR to induce host cell DNA synthesis is dependent on both the cell type and the status of contact inhibition.

## INTRODUCTION

The protozoan Toxoplasma gondii is an obligate intracellular pathogen that chronically infects approximately one-third of the human population (1). This widespread prevalence can be attributed, at least in part, to the successful manipulation of host defense mechanisms (2–4). Infection is typically initiated either through oral ingestion of tissue cysts from undercooked meat or oocysts that have been shed into the environment by infected felids, the definitive hosts of T. gondii (5). During the acute stage of infection, the rapidly dividing tachyzoite form of the parasite disseminates into multiple organs, including the immune-privileged regions of the body such as the central nervous system. Despite a robust mobilization of the adaptive immune response that resolves acute parasitemia, what remains behind, often undetected, are long-lived slow-growing tissue cysts (6, 7). As a result, in the United States more than 60 million people remain chronically infected by T. gondii, with disease typically manifesting in those whose immune systems become weakened or compromised (8, 9). Currently, the few drugs that effectively target this parasite are unable to cure chronic infection and thus allow for multiple rounds of reactivation in susceptible individuals (10).

T. gondii and other related members of the *Apicomplexan* phylum are defined by the presence of an apical complex structure that serves as a conduit to release, in a temporally regulated manner, the contents of three distinct apically targeted secretory organelles known as micronemes, rhoptries, and dense granules, which play a central role in parasite movement, invasion, and modulation of host cells (11–13). Over the last decade, researchers have shown that as a result of T. gondii infection, there is an active global reprogramming of host gene expression with distinct changes manifesting in pathways related to metabolism, transcriptional regulation, cell signaling, inflammation, and the cell cycle (14–16; reviewed in references 4, 17, 18). The parasite achieves this remarkable degree of cellular and organismal manipulation in part via an arsenal of secreted molecular effectors that it deploys against distinct host targets. Although considerable research efforts have highlighted the important role that the rhoptry secretory organelles play in parasite defense against the host, the dense granules (DG) have risen to a place of prominence as the source of numerous effectors that are critical for host manipulation by the parasite (19–25).

Of the many host transcriptional changes resulting from T. gondii infection, one of the most profound and consistent shifts is centered on the host cell cycle program itself (26–28). The extensively characterized eukaryotic cell cycle consists of four consecutive stages, abbreviated as G_1_, S, G_2_, and M. The G_1_/G_2_ gap (or growth) phases separate the DNA synthesis phase (S-phase) and M or mitotic phase, where the replicated genomes are divided into the new daughter cells prior to cytokinesis. The programmed advance of cells from one stage of the cell cycle to the next is tightly controlled by the phosphorylating activities of cyclins and their associated cyclin-dependent kinases (Cdk), which are, in turn, regulated by an extensive array of internal and external stimuli (29; reviewed in reference 30). Initial reports demonstrated that T. gondii infection induces a sustained increase in expression of mRNA transcripts association with the G_1_/S-phase transition (26), while ultimately arresting infected cells at the G_2_/M transition boundary (27). Two independent reports published in 2019 by Panas et al. and Braun et al. identified a host nuclear targeted dense granule protein, referred to both as HCE1, for inducer of host cyclin E (31), and TEEGR, for *Toxoplasma* E2F4-associated EZH2-inducing gene regulator (32), as being the parasite effector responsible. These reports also demonstrated that the HCE1/TEEGR effector binds to and activates the heterodimeric E2F/DP1 transcription factor complex, leading to the production of the cell cycle regulator cyclin E (31) while also activating the epigenetic silencer EZH2 to counteract the nuclear factor κB (NF-κB) proinflammatory response to parasite infection (32). In this study, we describe the independent identification of the HCE1/TEEGR effector based on the presence of distinct internal repeat regions commonly found in many previously identified nuclear targeted effectors of T. gondii (22–25, 33, 34). Our initial results serve to confirm prior observations that HCE1/TEEGR is a host nuclear targeted effector dense granule protein that induces distinct transcriptional signatures in host cells that highlight both the activation of the cell cycle program and suppression of NF-κB target genes. Our follow-up work, however, centers primarily on the kinetics of HCE1/TEEGR cell cycle manipulation and the ability of infected cells to transit through S phase. To interrogate the actions of this parasite effector on the cell cycle more closely, we implemented the fluorescence ubiquitination cell cycle indicator reporter line, known as FUCCI, and determined the distinct kinetics of S-phase transition that is dependent on both HCE1/TEEGR and the ability of T. gondii parasites to effectively traffic DG proteins across the parasitophorous vacuole (PV) membrane (35, 36). We observed that although infected human foreskin fibroblasts (HFF) and FUCCI (NIH 3T3) cells produced considerable levels of cyclin E and presented markers of S-phase transition, they were unable to progress through S-phase and synthesize new genomic DNA (gDNA) independent of the cell culture growth conditions. Newly derived primary mouse fibroblasts (MF), on the other hand, were able to progress through S-phase in an HCE1/TEEGR and contact inhibition-dependent manner. These data suggest that the ability of HCE1/TEEGR to drive infected host cells to transit through and complete S-phase is dependent on the cell line background as well as the status of contact inhibition.

## RESULTS

### HCE1/TEEGR (TGGT1_239010) is a host nuclear targeted dense granule protein requiring MYR1 for export.

**FIG 1:**
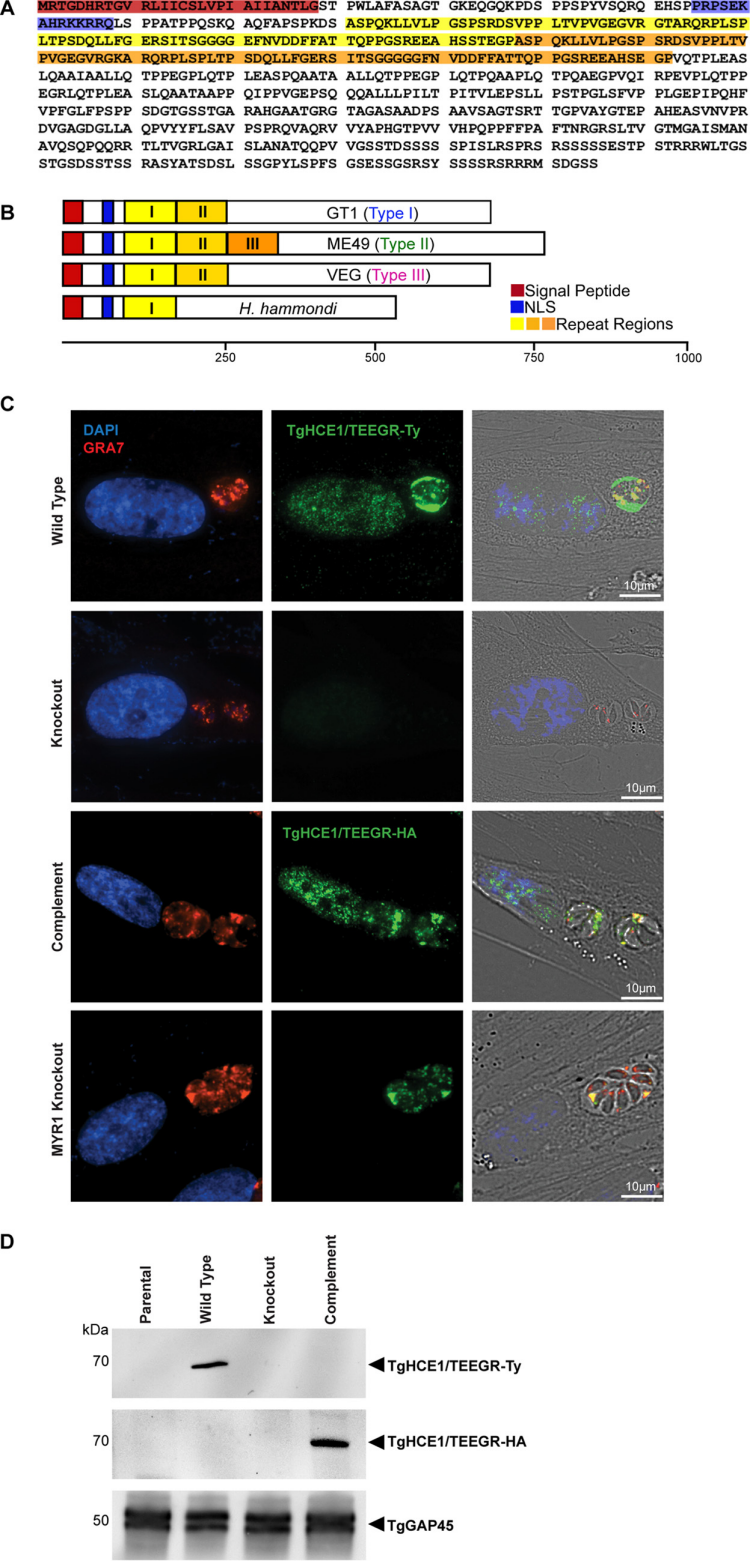
TgHCE1/TEEGR is a host nuclear targeted dense granule protein. (A) Amino acid sequence of TgHCE1/TEEGR in the GT1 type I strain displaying a predicted signal peptide in red, a nuclear localization signal (NLS) in blue, and two internal repeat sequences in yellow/orange. (B) Schematic representation of TgHCE1/TEEGR comparing type I, type II, and type III strains and the closely related species *H. hammondi*. The signal peptide (red), the nuclear localization signal (blue), and the differing numbers of repeated domains (light and dark orange) are highlighted. (C) HFF cells infected (20 h) with wild-type (TgHCE1/TEEGR-Ty), knockout (Tg*Δhce1/teegr-Ty*), complement (Tg*Δhce1/teegr-Ty*::HCE1/TEEGR-HA), and MYR1 knockout (Tg*Δmyr1*::HCE1/TEEGR-Ty) expressing T. gondii. HCE1/TEEGR (green), dense granule marker GRA7 (red), and DAPI nuclei (blue) are highlighted. Scale bar, 10 μm. Right, merge with bright field. (D) Western blot analysis of total infected host lysates confirming Ty epitope tagging of TgHCE1/TEEGR and Tg*Δhce1/teegr-Ty* and HA epitope tagging of TgHCE1/TEEGR in the complement.

To date, numerous reports have characterized, in detail, an array of secreted dense granule (DG) proteins of T. gondii that are targeted to the infected cell nucleus and directly modulate a variety of host transcriptional pathways. Of the host nuclear targeted DG effectors, such as GRA16, GRA24, GRA28, TgIST, and TgNSM, each published report noted the presence of internally repeated regions within these proteins that ranged from ~40 to 80 amino acids in length (22–25, 33, 34). Despite their seemingly ubiquitous presence in host nuclear targeted effectors, there has been no clear universal functional role ascribed to these repeats. The prevalence of this repeat pattern, however, suggested this is a common feature of nuclear targeted parasite proteins that could be used to identify novel DG effectors. To assess this possibility, we analyzed all T. gondii protein sequences containing predicted signal peptides (www.toxodb.org) using the online genome data-mining tool XSTREAM (https://amnewmanlab.stanford.edu/xstream/). This algorithm allowed for the broad identification of repeated regions in proteins ranging from a perfect match to highly degenerate. Using the XSTREAM program, we identified a hypothetical protein (TGGT1_239010) that contained a duplication of approximately 85 amino acids and a predicted nuclear localization signal (blue) (Fig. 1A and B). A phylogenetic analysis demonstrated the presence of this gene in the reference strains of T. gondii as well as the closely related Hammondia hammondi (see Fig. S1A in the supplemental material). Of note is the altered structure of the repeated regions, which potentially underwent several rounds of duplication with *H. hammondi* lacking these repeats, type I and III T. gondii strains containing a single duplication, and type II strains having three copies of this repeated region (Fig. 1B and Fig. S1B). Although the functional significance of the repeats remains unknown, this duplication suggests the presence of selective pressure to expand these regions. In prior published reports, this protein effector has been referred to as both HCE1, for inducer of host cyclin E (31), and TEEGR, for *Toxoplasma* E2F4-associated EZH2-inducing gene regulator (32), so they will be referred to collectively in this study as HCE1/TEEGR. By implementing a CRISPR/Cas9-based C-terminal Ty-tagging strategy using the parental type I RH *Δku80Δhxgprt* strain (37) of T. gondii (38, 39), we epitope tagged the *hce1/teegr* gene, referred to here as the wild-type (WT) strain. Through colocalization with the dense granule marker GRA7 (40–42), we confirmed previous work showing that HCE1/TEEGR-Ty is a DG protein that targets to the host cell nucleus (Fig. 1C, top). Because all secreted DG effectors previously observed to traffic to the host cell nucleus appear to require the action of the MYR translocon, we also tagged HCE1/TEEGR with a Ty epitope in the *Δmyr1* background and confirmed that the protein was no longer able to be transported across the PV into the host cell and thus failed to accumulate in the host nucleus (Fig. 1C, bottom) (31, 43). The epitope tagging of HCE1/TEEGR was also verified via Western blotting (Fig. 1D) and through diagnostic PCR (Fig. S1C) using the methods outlined in Fig. S1D.

10.1128/msphere.00160-22.1 FIG S1 Phylogenetic analysis of TgHCE1/TEEGR. (A) Phylogenetic analysis of HCE1/TEEGR in major T. gondii strains, including *H. hammondi*. (B) Phylogenetic analysis of the first repeat of the T. gondii strains, suggesting that the first repeat was the original domain prior to duplication. Specific alignment of the repeats from the type I domain likely occurred after strain divergence. Tree analysis of repeats from type I (blue) and type II (green) reveals that repeat 1 has fewer substitutions per site between strains (0.03) and is more similar between strains than repeat 1 and repeat 2 within strains (0.06 and 0.05). (C) PCR verification of genetic manipulations used for generating the wild-type, knockout and complemented strains. Primers for PCR are listed in Table S2. (D) Schematic describing the generation of wild-type, knockout, and complement lines in the RHΔku80Δhxgprt background. Download FIG S1, TIF file, 1.8 MB. Copyright © 2022 Pierre-Louis et al. 2022 Pierre-Louis et al. https://creativecommons.org/licenses/by/4.0/ This content is distributed under the terms of the Creative Commons Attribution 4.0 International license.

### HCE1/TEEGR promotes transcriptional changes to the host cell cycle program.

**FIG 2:**
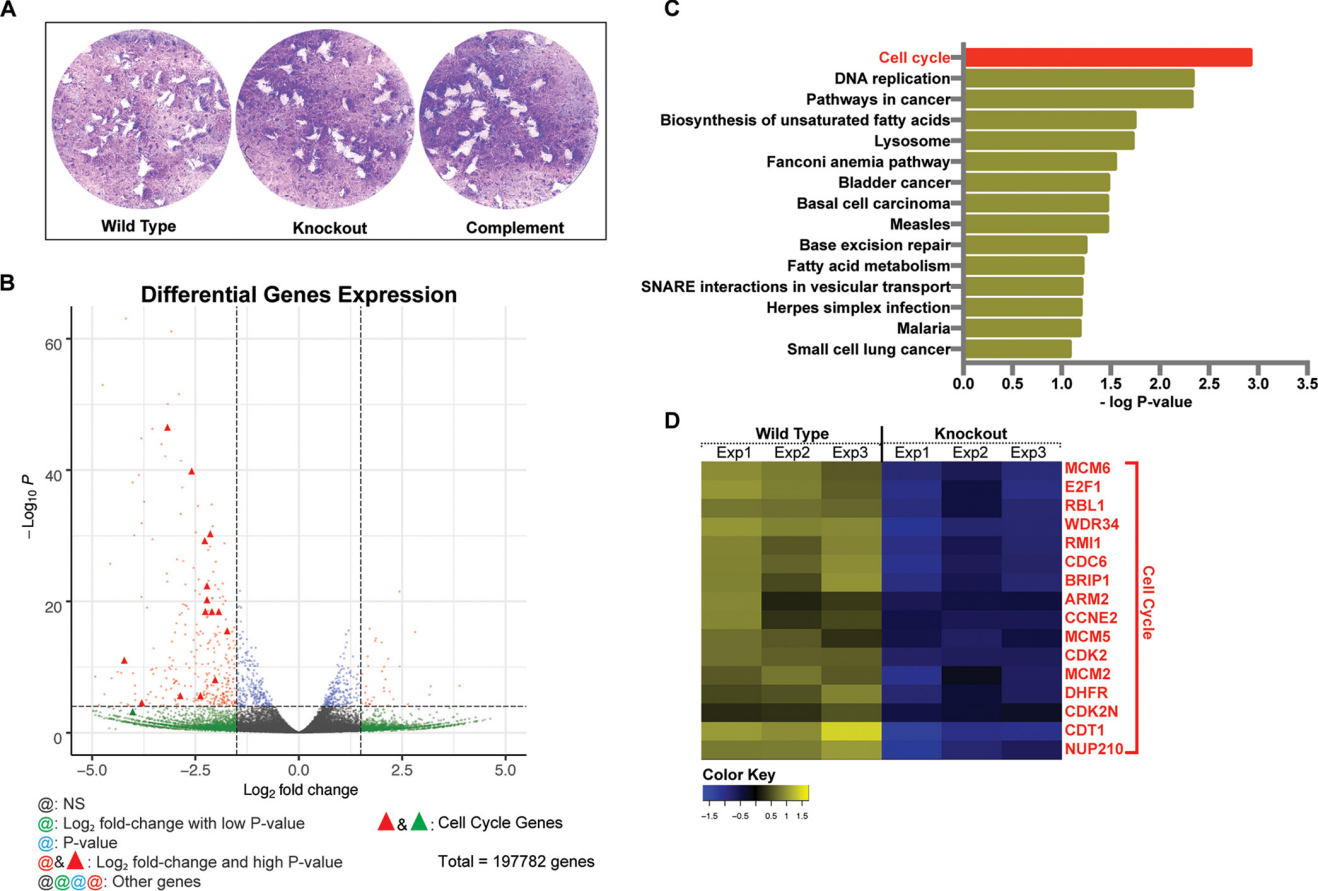
TgHCE1/TEEGR promotes activation of the host cell cycle program. (A) Plaque assays of wild-type (TgHCE1/TEEGR-Ty), knockout (Tg*Δhce1/teegr-Ty*), and complement (Tg*Δhce1/teegr-Ty*::HCE1/TEEGR-HA) tachyzoites on HFF monolayers. Each well was infected with 100 parasites, and the monolayers were fixed 7 days postinfection and stained with crystal violet. (B) Volcano plot illustration of RNA-Seq data depicting fold change of genes that are statistically significant in Tg*Δhce1/teegr-Ty*-infected HFFs showing upregulated genes (right quadrant/red dots) and downregulated genes (left quadrant/red dots and triangles). Red dots represent genes that have the highest *P* value with more than 1.5 log_2_ fold change. Red triangles are genes that are downregulated in Tg*Δhce1/teegr-Ty* (knockout) that are involved in the cell cycle. (C) Representation of top 94 differentially expressed genes that were identified with known pathway affiliations. Following the same parameters with transcripts showing a log_2_ fold change of >1.5 and representing a statistically significant differential expression (adjusted *P* value of <0.05), they were selected and classified into 15 pathways of the most upregulated and downregulated genes in TgHCE1/TEEGR-Ty- and Tg*Δhce1/teegr-Ty*-infected HFFs using DAVID6.8 and KEGG pathway analyses. (D) Differential expression of the top 16 genes that are upregulated (yellow) by wild type (TgHCE1/TEEGR-Ty) in infected HFF for 24 h and downregulated (blue) in knockout (Tg*Δhce1/teegr-Ty*) in infected HFF.

As a confirmation of prior published work, we generated gene deletion mutants in our epitope-tagged cell line (wild type) using CRISPR/Cas9-induced breaks in the *hce1/teegr* coding region followed by homology repair with the dihydrofolate reductase (DHFR) drug marker to generate a *Δhce1/teegr* knockout (KO) line (Fig. S1C and D, schematic) (38). The deletion mutant no longer contained the *hce1/teegr* gene as verified using diagnostic PCR (Fig. S1C), resulting in loss of expression as depicted in the Western blot and immunofluorescence microscopy (IFA) of infected cells (Fig. 1C and D). As expected, HCE1/TEEGR deletion resulted in no significant defect in parasite growth *in vitro*, as demonstrated in our plaque assays (Fig. 2A), or virulence in CD1 mice (Fig. S2A). Additionally, we conducted a whole-transcriptome sequencing experiment (RNA-Seq) of HFFs comparing host cells infected with parasites expressing HCE1/TEEGR-Ty (WT) to those in which the *hce1/teegr* gene had been deleted (knockout). HFFs were infected for 16 h at a multiplicity of infection (MOI) of 5:1, followed by total RNA isolation and Illumina-based sequencing. The resulting data (Fig. 2B to D) confirmed that HCE1/TEEGR was responsible for both the upregulation of pathways associated with the host cell cycle as well as a downregulation of genes associated with the nuclear factor κB (NF-κB) proinflammatory response (Fig. S2B) (31, 32). Figure 2B highlights genes that were significantly downregulated (red dots in left quadrant) in the *Δhce1-teegr*-infected host cells, with the red triangles representing specific genes with known involvement in controlling the host cell cycle (reviewed in reference 30). Using DAVID6.8 and KEGG pathway analysis, we examined the top 94 genes with the highest differential expression that were affiliated with known cellular pathways (Fig. 2C) and confirmed an enrichment in pathways associated with “Cell cycle” and “DNA replication.” In examining the top 16 genes that were significantly downregulated across the three replicates (Fig. 2D), we also observed a concentration of genes involved in cell cycle progression. As described in the Panas et al. study (31), we confirmed an upregulation of cyclin E (CCNE2) and its associated cyclin-dependent kinase (CDK2) as well as the machinery involved in origin licensing (e.g., CDT1, CDC6, and MCM2/6), all of which confirmed a role for HCE1/TEEGR in promoting a cellular transition into S-phase (44–46). HCE1/TEEGR’s capacity to drive host cell cycle gene expression was elegantly shown in prior studies to be due to its interaction with the E2F/DP1 heterodimer, leading to induction of cell cycle gene expression (31) while also activating the epigenetic silencer EZH2, which modulates the NF-κB proinflammatory response to T. gondii infection (32).

10.1128/msphere.00160-22.2 FIG S2 Impact of TgHCE1/TEEGR on mouse virulence and global gene expression in infected host cells. (A) Virulence in the mouse model of infection. Mice were injected intraperitoneally (IP) with 100 T. gondii cells and monitored for survival. Survival curve for CD1 mice infected with wild-type and knockout parasites over 9 days. (B) Global impact analysis of differentially expressed genes comparing both significance and fold change of upregulated and downregulated genes during wild-type and knockout infections. With upregulated genes in yellow and downregulated in blue. Three biological replicates are shown. Download FIG S2, TIF file, 1 MB. Copyright © 2022 Pierre-Louis et al. 2022 Pierre-Louis et al. https://creativecommons.org/licenses/by/4.0/ This content is distributed under the terms of the Creative Commons Attribution 4.0 International license.

### HCE1/TEEGR induces infected host cells into S-phase.

**FIG 3:**
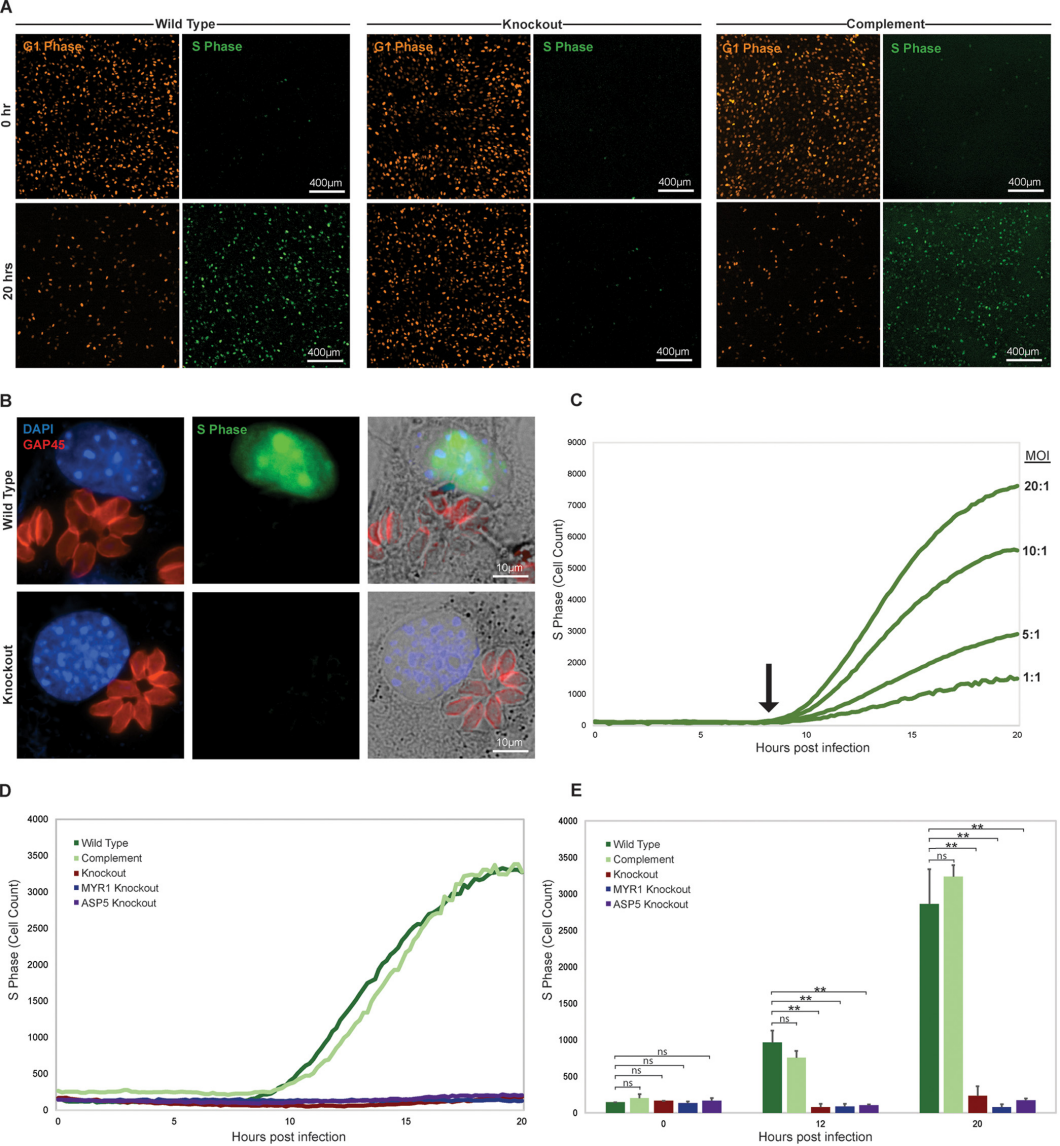
TgHCE1/TEEGR drives infected host cells into S-phase. (A) FUCCI cells infected with wild-type (TgHCE1/TEEGR-Ty), knockout (Tg*Δhce1/teegr-Ty*), and complement (Tg*Δhce1/teegr-Ty*::HCE1/TEEGR-HA) strain at 0 (top rows) and 20 (bottom rows) h postinfection. G_1_ phase (left column) and S-phase (right column) of the same field of FUCCI cells are shown. Scale bar, 400 μm. (B) FUCCI cells infected (20 h) with TgHCE1/TEEGR-Ty (top row) and Tg*Δhce1/teegr-Ty* (bottom row) expressing T. gondii. GAP45 parasite marker (red), DAPI nuclei (blue), and S-phase FUCCI (green) are shown. Scale bar, 10 μm. Right, merge with bright field. (C) Multiplicity of infection (MOI) ratios for TgHCE1/TEEGR-Ty-infected FUCCI cells over 20 h. Ratios shown are 20:1, 10:1, 5:1, and 1:1 (T. gondii to FUCCI cell). (D and E) G_1_ to S-phase conversion of FUCCI cells infected with wild type (TgHCE1/TEEGR-Ty) in dark green, knockout (Tg*Δhce1/teegr-Ty*) in red, complement (Tg*Δhce1/teegr-Ty*::HCE1/TEEGR-HA) in light green, MYR1 knockout (Tg*Δmyr1*) in blue, and ASP5 knockout (Tg*Δasp5*) in purple. Results are representative of three experimental replicates quantified in panel E. Cell count represents total green nuclei in host cells. Statistical analysis was done using a Student's *t* test. *, *P* < 0.05; **, *P* < 0.01; ns, not significant.

One of the major checkpoints in the transition of mammalian cells into S-phase is the firing of origin replication complexes (ORC), which initiates the process of gDNA synthesis and genome duplication (47, 48). This event results from the buildup, during G_1_, of active cyclin E/CDK2 complexes and the loading of replication machinery at the origins by Cdt1 (46). The subsequent firing of ORCs and the initiation of gDNA replication is immediately followed by rapid destruction of Cdt1 (G_1_ marker) and the buildup of the Cdt1 inhibitor protein Geminin (S-phase marker) to prevent the reinitiation of replication (49). The cyclical buildup and destruction of Cdt1 and Geminin has been shown to be mediated via their interaction with distinct F-box proteins and associated ubiquitin ligases that control the abundance of these S-phase regulators by inducing proteasomal degradation (reviewed in reference 50). Using this information, several groups have fused the F-box targeting domain of Cdt1 and Geminin to fluorescent proteins to observe transit through the stages of the cell cycle in live cells (51). Commonly referred to as FUCCI, for fluorescence ubiquitination cell cycle indicator, these reporters have been integrated in a variety of cell lines and whole-animal models to examine factors (e.g., cell type, genes, growth factors, and chemical agents) that influence the progression of cells through the cell cycle (35, 36). To observe the effects of T. gondii infection on the host cell cycle directly, we used a FUCCI cell cycle reporter line generated via the transfection of immortalized mouse embryonic fibroblast (NIH 3T3) cells with a FUCCI-expressing lentivirus (a generous gift from Jonathan Eggenschwiler). In this FUCCI cell line, the red fluorescent reporter represents Cdt1 levels (G_1_ phase), while green fluorescence serves as a stand-in for Geminin (S-phase). We infected confluent monolayers of FUCCI reporter cells arrested in G_0_/G_1_ with wild-type (HCE1/TEEGR-Ty tagged), knockout (*Δhce1-teegr-Ty*), and complemented (*Δhce1/teegr-Ty*::*HCE1/TEEGR-HA*) parasites at an MOI of 5:1 and observed the host monolayer fluorescence at 20 h postinfection. Through examining the fluorescence of infected host nuclei, we observed a clear transition of the majority of FUCCI cells in the monolayer from the G_0_/G_1_ phase (red) into S-phase (green) in both the wild-type and complemented line infections (Fig. 3A, left and right). Importantly, we failed to observe any significant fluorescence transition in FUCCI cells infected with the *Δhce1/teegr* knockout line (Fig. 3A, middle). To examine the dependence of S-phase transition on HCE1/TEEGR at the single-cell level, we stained infected FUCCI cells with antibodies to the parasite protein GAP45 to highlight intracellular T. gondii and observed that the induction of the green fluorescent S-phase nuclear reporter occurred only in cells infected with wild-type parasites (Fig. 3B top) and not in the knockout infection (Fig. 3B, bottom) (52). The exact kinetics of this transition to S-phase and the dose dependence of HCE1/TEEGR on this transition, however, were still unclear, since our original observations relied only on a single time point (20 h) and MOI (5:1). We next infected FUCCI cells with a range of MOIs (1:1, 5:1, 10:1, and 20:1) and assessed, in real time, the rate of transition into S-phase following infection. Using automated time course live-cell microscopy, we assessed the kinetics of S-phase transition in host cells infected with increasing MOIs of wild-type T. gondii parasites. Over a period of 20 h, we acquired images of infected FUCCI cells at 10-min intervals and counted the total number of host cell nuclei expressing green fluorescent protein as a readout of S-phase transition (Fig. 3C). We observed that although the total number of FUCCI cells that transitioned into S-phase was MOI dependent and likely a function of infection rate, the timing of transition into S-phase was independent of MOI and occurred at approximately 8 h postinfection (Fig. 3C, arrow). Therefore, because of the consistency in the kinetics of S-phase induction, we reason that the quantity of secreted HCE1/TEEGR from an individual wild-type parasite is sufficient to promote S-phase transition at the maximum rate. To validate the dependence on S-phase transition kinetics of HCE1/TEEGR, we infected FUCCI cells with wild-type, knockout, and complemented lines as well as *Δmyr1* and *Δasp5* knockout lines, which are known to be defective in DG protein translocation and processing, respectively (43, 53). When *Δhce1/teegr* parasites were allowed to infect FUCCI cells, no S-phase transition was observed by immunofluorescence, and this phenotype was restored in the complemented line with kinetics similar to those of the wild-type parental strain (Fig. 3D). Additionally, the loss of MYR1 or ASP5 phenocopied the loss of HCE1/TEEGR, further confirming their role in the localization and maturation of this DG protein. A quantitative analysis and comparison of the number of host cells in S-phase at three time points (0, 12, and 20 h postinfection) using these different parasite strains demonstrated a clear dependence on secreted HCE1/TEEGR for the capacity of T. gondii infection to drive FUCCI cells into S-phase (Fig. 3E). These observations are consistent with prior studies of parasites lacking HCE1/TEEGR or the ability to effectively secrete dense granule proteins, being unable to promote cell cycle changes in infected cells (31).

### HCE1/TEEGR promotes S-phase progression with increased cyclin E production.

**FIG 4:**
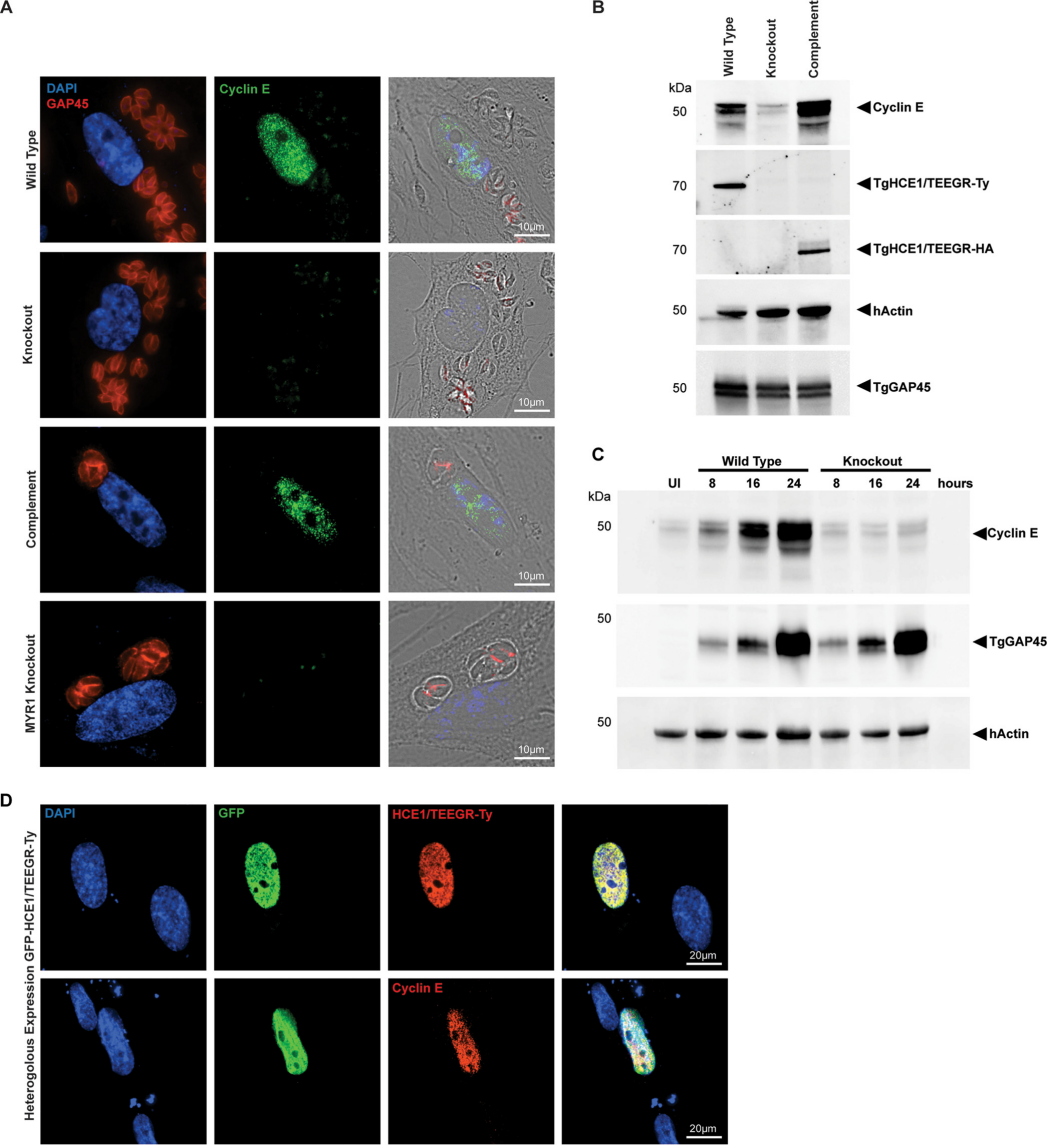
TgHCE1/TEEGR induces production of host cyclin E. (A) HFF cells infected (24 h) with wild-type (TgHCE1/TEEGR-Ty), knockout (Tg*Δhce1/teegr-Ty*), complement (Tg*Δhce1/teegr-Ty*::HCE1/TEEGR-HA), and MYR1 knockout expressing HCE1/TEEGR-Ty (Tg*Δmyr1*::HCE1/TEEGR-Ty) T. gondii. Cyclin E (green), GAP45 parasite (red), and DAPI nuclei (blue) are shown. Scale, 10 μm. Right, merge with bright field. (B) Western blot analysis of cyclin E expression from wild-type (TgHCE1/TEEGR-Ty), knockout (Tg*Δhce1/teegr-Ty*), and complement (Tg*Δhce1/teegr-Ty*::HCE1/TEEGR-HA) strain-infected HFF cells after 24 h. (C) Western blot analysis of cyclin E expression from uninfected (UI) as well as wild-type (TgHCE1/TEEGR-Ty) and knockout (Tg*Δhce1/teegr-Ty*) strain-infected HFF cells. Nuclear lysates were collected at 8, 16, and 24 h postinfection. (D) HFF cells transfected with pGFP-HCE1/TEEGR-Ty (24 h). DAPI nuclei (blue), GFP (green), cyclin E (red, top), and Ty-epitope (red, bottom) are shown. Scale bar, 20 μm. Right, merge.

As observed in both prior reports on HCE1/TEEGR effects on the host cell cycle (31) and our RNA-Seq analysis, the transcription of cyclin E (CCNE2) is highly upregulated during T. gondii infection. This cyclin plays a well-studied and critical role in the G_1_- to S-phase transition in replicating cells through its association with and activation of the cyclin-dependent kinase CDK2 (reviewed in references 54 and 55). To confirm cyclin E production at the protein level, we examined human foreskin fibroblasts (HFF) infected with either wild-type, knockout, HCE1/TEEGR complemented, or *Δmyr1* knockout strains and assessed the relative change in cyclin E production after 24 h of infection. We validated the dependence of HCE1/TEEGR export for T. gondii parasites (red) to be able to induce the production of nuclear targeted cyclin E (green) in infected HFFs (Fig. 4A). As seen previously, parasite strains lacking HCE1/TEEGR (knockout) or unable to export HCE1/TEEGR into the host cell (MYR1 knockout) were not able to induce cyclin E expression, while the complemented line demonstrated robust nuclear expression of this critical cell cycle regulator. The HCE1/TEEGR-dependent induction of cyclin E production was also confirmed using Western blotting at 24 h postinfection (Fig. 4B). The kinetics of cyclin E induction was also demonstrated in a time course analysis of cyclin production at 8, 16, and 24 h postinfection (Fig. 4C). The use of antibodies to GAP45 highlighted the expanding number of replicating parasites across the time course, while antibodies to the host actin protein (hActin) were used to ensure equal loading of uninfected (UI) and infected host cell material. The increase in parasite GAP45 levels in both strains supports our prior observations that a lack of HCE1 does not significantly impact parasite growth *in vitro* (Fig. 2A, plaque assay). Finally, we wanted to confirm that HCE1/TEEGR alone was indeed sufficient to induce cyclin E expression. To achieve this, we, like others, conducted a heterologous expression experiment where we transfected HFF cells with an overexpression vector containing HCE1/TEEGR-Ty with GFP fused to its N terminus as a test of sufficiency. We noted the localization of this protein fusion product in the nucleus of transfected cells, observing green fluorescence from the GFP N-terminal fusion and also labeling of the Ty epitope, which is fused to the C terminus of HCE1/TEEGR (Fig. 4D, top). We next assessed whether or not the expression of the HCE1/TEEGR-Ty fusion protein in HFFs also activated cyclin E production and observed that all HFF cells expressing HCE1/TEEGR (green) also showed increased expression of cyclin E (red) at 24 h posttransfection (Fig. 4D, bottom). This work, therefore, confirmed prior observations that HCE1/TEEGR alone is sufficient to drive the production of cyclin E and thus an activation of the cell cycle transition into S-phase (31).

### Infected HFF and FUCCI cells fail to progress through S-phase.

**FIG 5:**
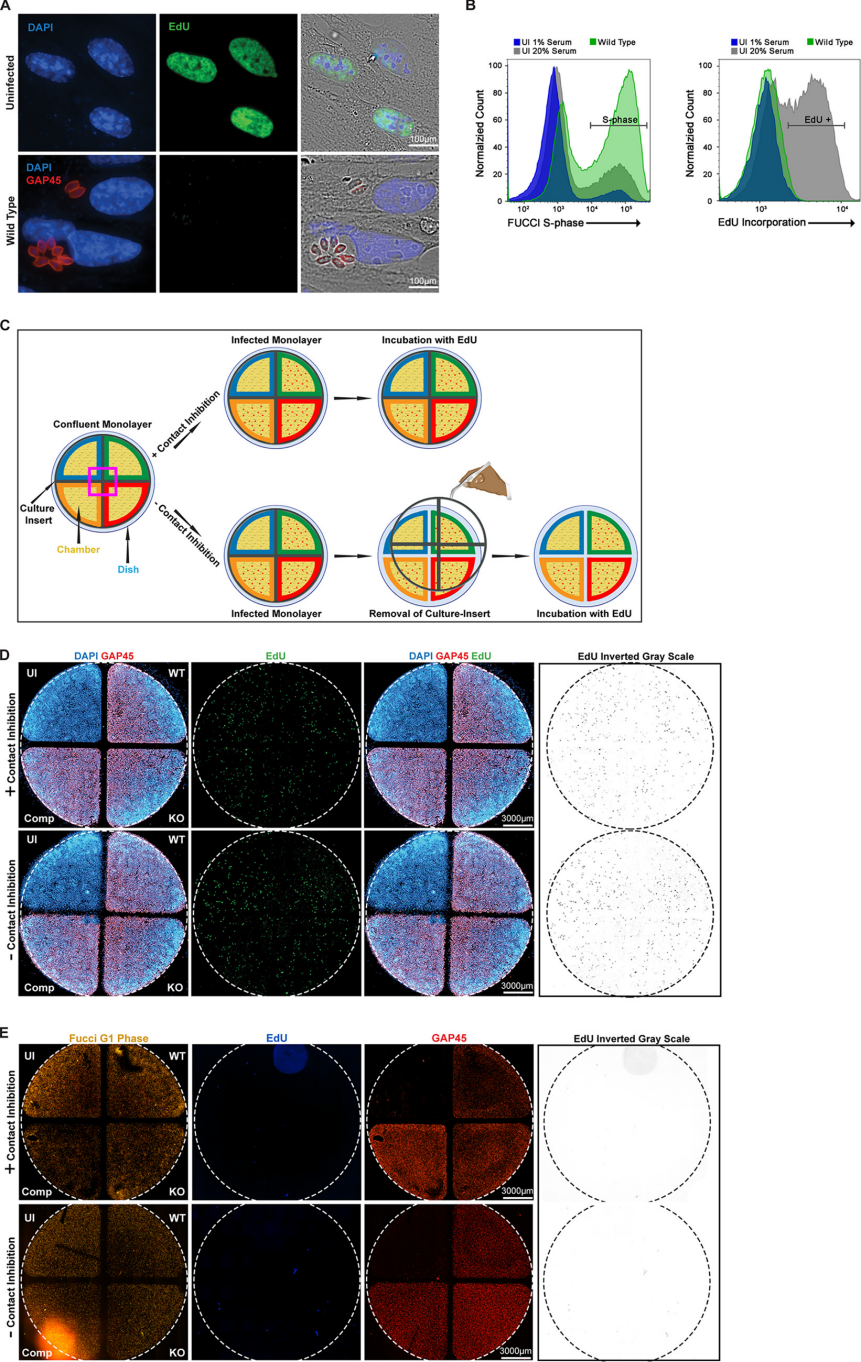
Infected HFF and FUCCI cells in S-phase are unable to synthesize new DNA. (A) HFF cells were incubated with EdU and either left uninfected or infected with wild type (TgHCE1/TEEGR-Ty). Monolayers were fixed after 24 h and stained for EdU incorporation using Alexa Fluor 488 (green), parasites using α-GAP45 (red), and nuclei using DAPI (blue). Scale bar, 100 μm. (B) Histogram of FUCCI cells analyzed by flow cytometry. Cells were incubated with EdU and either uninfected, grown in 1% or 20% serum, or infected with wild-type (TgHCE1/TEEGR-Ty) T. gondii. Cells were fixed at 24 h and labeled with EdU and Alexa Fluor 405. (C) Schematic displaying the procedure for the wound healing assay to assess contact inhibition. Pink box represents area of zoom. (D and E) Wound healing assay either in the presence (+ contact inhibition) or absence (− contact inhibition) of contact inhibition using HFF (D) or FUCCI (E) cells. Cells were either left uninfected (UI) or infected with wild-type (TgHCE1/TEEGR-Ty), knockout (Tg*Δhce1/teegr-Ty*), or complement (Tg*Δhce1/teegr-Ty*::HCE1/TEEGR-HA) lines. Monolayers were then incubated for 24 h with EdU in medium containing 1% serum. The areas to the right in panels D and E represent the inverted gray scale of the original EdU from panels D and E. Graphs represent three biological replicates. Scale, 3,000 μm.

Prior reports on the ability of T. gondii infection to promote host cells to enter into the cell cycle have, with few exceptions, concluded that infection leads to progression through S-phase with arrest in G_2_/M and with infected cells failing to undergo mitosis and cytokinesis (26, 43, 56). To address this aspect of the cell cycle progression, we examined the ability of infected cells to replicate their genome via the incorporation of the alkyne-containing thymidine analogue 5-ethynyl-2′-deoxyuridine (EdU) and its subsequent detection by a fluorescent azide reporter through a copper-catalyzed click chemistry reaction (57). Using actively dividing HFFs (i.e., subconfluent monolayer growing in 20% serum) as a positive control, we saw clear incorporation of EdU (green), highlighting the ability of these cells to synthesize new gDNA as part of their normal replication program (Fig. 5A, top). Interestingly, we found that when confluent HFFs were infected with wild-type T. gondii, we observed no evidence of cells being able to transit through S-phase and incorporate EdU in spite of an abundance of S-phase cyclins being produced in response to infection (Fig. 5A, bottom). Using HFFs, we could not rule out the possibility that although they were producing high levels of cyclin E, as seen throughout Fig. 4, they, unlike the FUCCI cells, were unable to fire their origins of replication and actually enter S-phase. To examine this further, we decided to use flow cytometry to observe gDNA replication directly in FUCCI cells that had been infected with wild-type parasites. We compared uninfected confluent G_0_/G_1_-arrested FUCCI cells (UI, 1% serum) to both actively growing uninfected FUCCI cells and FUCCI cells infected with wild-type parasites (Fig. 5B, left). As expected, a significant proportion of actively growing uninfected (UI, 20% serum) and parasite-infected FUCCI cells transitioned into S-phase, as demonstrated by the accumulation of green fluorescence signal in these cells. We next gated solely on those FUCCI cells that had transitioned into S-phase (green) and examined the level of EdU incorporation present in these S-phase cells. Curiously, we observed that only the uninfected, actively growing FUCCI cells (UI, 20% serum) appeared to be synthesizing new gDNA (Fig. 5B, right, gray population). Thus, much like our observations in HFF cells (Fig. 5A), there was no detectable gDNA replication in the infected FUCCI cells despite a clear transition into S-phase. This suggested that although the infected cells fired their origins of replication and increased expression of the S-phase marker Geminin (green), they were blocked in their ability to effectively progress through this stage and replicate their genomes.

As has been described in multiple cell cycle studies, there are secondary control systems in place to block the ability of mammalian cells to progress through S-phase despite an abundance of necessary cyclin-Cdk complexes and growth stimuli. These negative regulation mechanisms are varied and can be activated by DNA damage, stress, or contact inhibition (58–60). Although there are reports that T. gondii infection can induce DNA damage in infected host cells (61), we initially suspected that contact inhibition, driven in part by the production of CDK inhibitors p27 (Kip1), was responsible for this block in S-phase progression. To examine the effect of contact inhibition on this phenomenon more closely, we employed techniques pioneered by groups studying fibroblast wound healing to assess the effect of release from contact inhibition on S-phase progression (62, 63). As depicted in Fig. 5C, we used a four-chamber 35-mm cell culture dish containing a removable four-quadrant insert for our assay. When removed, the resulting scar breaks contact inhibition on the monolayer and stimulates the entrance of cells into the barren region between quadrants to reestablish confluence. The use of these four chambered culture dishes allowed us to also ensure intraexperimental consistency as the host monolayer in each dish was subjected to the same medium and growth conditions. For simplicity, we have color-coded the experimental conditions applied to each of the four regions. Beginning in the top left quadrant and moving clockwise, we have the uninfected host cell control (UI) (blue), followed by the wild-type (green), the *Δhce1/teegr* knockout (KO) (red), and the HCE1/TEEGR complemented (Comp) infections (orange). We first sought to examine the potential effect of release of contact inhibition on S-phase progression and EdU incorporation using both our HFF and FUCCI cell lines in this four-quadrant wound healing assay format. Using HFF cells, we observed that irrespective of the infection status and whether the insert was present (Fig. 5D, top, + contact inhibition) or removed (Fig. 5D, bottom, − contact inhibition), these cells could not effectively progress through S-phase and incorporate EdU. Using this same experimental setup applied to FUCCI cells (Fig. 5E), we again found no effect of contact inhibition on S-phase progression at 20 h postinfection. Cells incorporating EdU are highlighted in the inverted grayscale panel to the right in Fig. 5D and E. Similar to wounding the monolayer, studies have demonstrated that splitting and replating the cells at lower density supplemented with fresh serum can quickly reinstate proliferation in contacted-inhibited cells (60). However, when we employed this technique on infected HFF cells that were trypsinized, removed from the dish, and replated in 20% serum, they, unlike uninfected HFF cells, failed to actively synthesize gDNA (Fig. S3A and B). This suggested the presence of either a parasite-driven mechanism blocking S-phase progression or an unknown cell-intrinsic mechanism. We repeated the replating experiment using *Δmyr1* parasites and again observed no return to S-phase progression, with EdU incorporation rates remaining unchanged, making it unlikely that a dense granule effector was blocking gDNA synthesis (Fig. S3C) (43, 64).

10.1128/msphere.00160-22.3 FIG S3 TgHCE1/TEEGR does not activate DNA replication when infected host cells are replated at subconfluent densities. (A and B) HFF cells were either uninfected or infected with wild-type or KO T. gondii for 2 h. Monolayers were then replated and seeded subconfluent and incubated with EdU in media containing 20% serum. Top panel represents uninfected cells that were replated as positive control. Parasites were stained with GAP45 (red), host nuclei with DAPI (blue), and replicating DNA with EdU (green). Quantitation composed of three biological replicates. Statistical analysis was done using a Student’s *t* test. *, *P* < 0.05; **, *P* < 0.01; ***, *P* < 0.01; ns, not significant. Scale, 300 μm. (C) HFF cells were infected with Tg*Δmyr1*::HCE1/TEEGR-Ty for 2 h. Monolayers were then replated, seeded subconfluently, and incubated with EdU in media containing 20% serum. Scale, 300 μm. Download FIG S3, TIF file, 2.8 MB. Copyright © 2022 Pierre-Louis et al. 2022 Pierre-Louis et al. https://creativecommons.org/licenses/by/4.0/ This content is distributed under the terms of the Creative Commons Attribution 4.0 International license.

### Contact inhibition is required to block S-phase progression in primary mouse fibroblasts.

**FIG 6:**
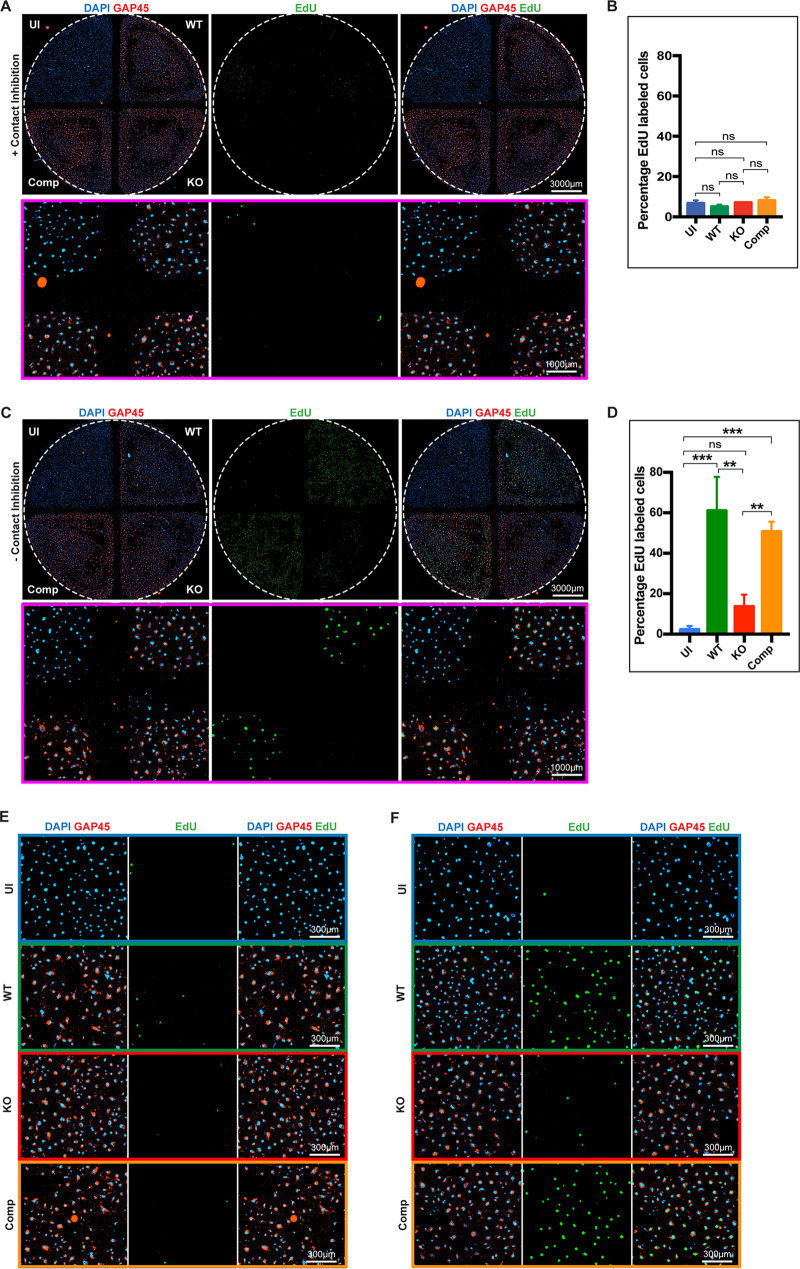
TgHCE1/TEEGR induces S-phase DNA replication in primary mouse fibroblasts upon removal of contact inhibition. (A) Wound healing assay using mouse fibroblasts (MF) in the presence of contact inhibition. Host cell DNA was stained with DAPI (blue), and parasites were stained with GAP45 (red) and EdU (green). (Top) View of all 4 quadrants with uninfected (UI), infected WT (TgHCE1/TEEGR-Ty), KO (Tg*Δhce1/teegr-Ty*), or Comp (Tg*Δhce1/teegr-Ty*::HCE1/TEEGR-HA) parasite lines (scale, 3,000 μm). (Bottom) Zoom of central junction of 4-quadrant dish (scale, 1,000 μm). (B) Quantitation of 3 biological replicates of panel A. (C) Wound healing assay as in panel A with contact inhibition removed. (Top) All quadrants (scale, 3,000 μm). (Bottom) Zoom of central junction of 4-quadrant dish (scale, 1,000 μm). (D) Quantitation of 3 biological replicates of panel C. (E) Zoom of central portion of each quadrant (A, + contact inhibition) (scale, 300 μm). (F) Zoom of central portion of each quadrant (C, − contact inhibition) (scale, 300 μm). Confluent monolayers were then incubated for 20 h with EdU in media containing 1% serum. Graphs represent three biological replicates comparing the different conditions in the presence or absence of contact inhibition. Statistical analysis was done using one-way ANOVA. *, *P* < 0.05; **, *P* < 0.01; ***, *P* < 0.001;  ns, not significant.

The cell lines we examined thus far were either high-passage-number primary lines (HFF) or immortalized (FUCCI NIH 3T3), so we decided to examine the ability of parasites to induce progression through S-phase in recently isolated MF. We first isolated primary MFs from C57BL/6 mouse tissue, grew cells to confluence in each quadrant of the four-chamber dish, and subjected each quadrant to conditions analogous to those previously described (Fig. 5C to E). Infection was allowed to progress for 20 h in the presence of EdU in 1% serum before analysis. In the initial control experiment, the chamber insert was retained to preserve contact inhibition, and we observed that regardless of the parasite strain or conditions used during infection, no increase in incorporation of EdU was seen (Fig. 6A, top). Focusing at a higher magnification on the center of the four-quadrant junction (Fig. 6A, bottom), we can assess the level of T. gondii infection using the parasite-specific GAP45 antibody (red) and the presence of 4′,6-diamidino-2-phenylindole (DAPI)-stained host cell nuclei (blue) (52). A quantitative analysis demonstrated no significant change in EdU incorporation irrespective of the infection conditions or parasite strain used (Fig. 6B). To assess the role of contact inhibition, we again infected each chamber of confluent cells with their respective strains as before; however, directly following infection we removed the chamber insert (− contact inhibition) and allowed the infection to proceed overnight in the presence of EdU and 1% serum. At 20 h postinfection we fixed and stained cells and examined the level of EdU incorporation (Fig. 6C, top) with a focus on the central junction. Under these conditions, we observed a striking increase in EdU-positive cells (green) in both the WT- and Comp-infected quadrants when contact inhibition was removed and failed to see comparable changes in either the UI or *Δhce1/teegr* KO-infected monolayers (Fig. 6C, top). This difference is also clearly illustrated when we focus on the central junction of the plate to highlight each quadrant (Fig. 6C, bottom). Quantitative analysis of three independent replicates demonstrated a significant increase in the percentage of cells synthesizing new gDNA in the WT- and Comp-infected MFs with no significant change in the UI or KO-infected quadrants (Fig. 6D). To visually highlight the dramatic differences observed, we also examined central regions of each quadrant in both the presence (Fig. 6E) and absence (Fig. 6F) of contact inhibition, with each quadrant color-coded to match the starting quadrant. From this work, it appears that a lack of gDNA replication observed in T. gondii-infected cells could result from either a cell line-specific block, as seen in HFFs and NIH 3T3 cells, or contact inhibition, as seen with MF infections.

## DISCUSSION

The work presented here not only confirms and supports many of the prior published observations concerning the action of the HCE1/TEEGR effector but also furthers our understanding of how T. gondii manipulates the host cell cycle in distinct ways. In our transcriptome analysis, we confirmed the ability of this nuclear targeted dense granule effector to drive both the transcriptional activation of the cell cycle program as well as downregulation of NF-κB proinflammatory genes (31, 32). Additional experiments also validated prior observations showing that HCE1/TEEGR was sufficient to induce a dramatic increase in host protein levels of cyclin E over time (31). Due to the prominence of the cell cycle pathways activated by HCE1/TEEGR, and because multiple prior studies had described this phenomenon, we focused our work primarily on dissecting HCE1/TEEGR’s ability to manipulate this host process in more detail. To assess changes in the host cell cycle by HCE1/TEEGR, we implemented the FUCCI cell cycle reporter line to investigate the kinetics of S-phase transition after infection. We were led to this option after repeated attempts to assess the host cell cycle status using standard DNA staining and flow cytometry failed to produce clear distinctions between the major stages (G_1_, S-phase, and G_2_). We suspect that the DNA content contributed by growing parasites within infected cells was confounding our ability to accurately assess changes in host DNA content. Our examination of the infected host cell cycle using FUCCI cells, therefore, allowed us to directly measure the timing of transition into S-phase that could be looked at across an infection time course. From these experiments, we were able to pinpoint the approximate timing of S-phase transition after the beginning of infection (approximately 8 h) and realized that this timing could not be accelerated by increasing the multiplicity of infection of individual host cells. This suggested that the amount of HCE1/TEEGR secreted by a single invaded tachyzoite is sufficient to induce host cell transition into S-phase at the maximal rate. In our continued analysis, however, we noted that despite the assumption that infected cells are blocked in G_2_/M, we never observed host nuclei that appeared enlarged or even in an intermediate stage of mitosis. We assumed that if S-phase initiated at 8 h postinfection, we should be seeing cells at some stage of mitosis 20 h postinfection even if cytokinesis was being hindered by parasite replication and potential steric hindrance. To look at this more closely, we used the click-chemistry-amenable thymidine analogue EdU to visualize gDNA synthesis (65). Curiously, we found that infected HFF cells failed to incorporate EdU at levels we could measure either using microscopy or flow cytometry and thus appeared blocked in their ability to progress through S-phase. We were unsure, however, if infected HFFs were in fact transitioning fully into S-phase. Conveniently, the FUCCI cells allowed us to resolve this issue, since we had already observed that, when infected, these cells produce markers of S-phase, as evidenced by the destruction of their reporter of Cdt1 (red) and the accumulation of the Geminin reporter (green). These fluorescent reporters gave us a window into the process of prereplication complex destruction that occurs following the initiation of gDNA replication (51). Unexpectedly, even when pushed to initiate S-phase, the FUCCI line (mouse NIH 3T3) failed to synthesize new gDNA, mirroring the observations from infected HFF cells. Our first concern was that this may be due, in part, to the negative feedback signals on cell cycle progression, such as contact inhibition (29). As a result, we conducted a hybrid infection/wound healing assay to produce a controlled release from contact inhibition in infected cells. We surmised that following such release, this inhibitory signal would be removed and, therefore, should allow gDNA replication to proceed (62, 66). Instead, we found that breaking contact inhibition did not rescue gDNA replication even when infected cells were replated at a subconfluent concentration under conditions normally supporting active growth. Because prior work examining other DG proteins such as GRA16 and GRA24 had demonstrated that these effectors manipulate both the tumor suppressor protein p53 and the mitogen-activated kinase signaling pathway p38, respectively, it seemed plausible that additional DG effectors could be contributing to the block in gDNA synthesis (24, 25). We suspected that T. gondii was inducing entrance into S-phase with HCE1/TEEGR while also blocking progression through S-phase via an additional unidentified effector. However, we observed that even when HFF cells were infected with the *Δmyr1* strain of T. gondii, they were unable to initiate gDNA replication even when replated under active growing conditions (43). This suggested that the gDNA replication block we observed was not due to the presence of an additional secreted DG protein effector, and the fact that all of the cancer lines we have tested can progress through S-phase and replicate their genomes argued against a rhoptry effector being responsible (see Fig. S4A and B in the supplemental material). Other possible reasons for the block in S-phase progression were the presence of cell cycle inhibitors being activated due to host DNA damage and some other infection-associated stresses. The first potential cell cycle regulators we sought to investigate were the DNA damage-responsive cyclin-Cdk inhibitor p21 (Cip) and the contact inhibitor p27 (Kip) (67–70). Due to the commercial availability of homozygous p21^−/−^ knockout mice, we isolated primary fibroblasts (MF) from both wild-type and p21^−/−^ C57BL/6 mice to examine the role of p21 in the observed block in gDNA replication in T. gondii-infected cells (71). We first set out to establish the assay methodology and confirm that wild-type MFs would be unable to replicate their gDNA following infection, as seen in HFFs and FUCCI cells. When confluent monolayers of wild-type MFs were infected, we observed, as expected, an inability of these cells to incorporate EdU and progress through S-phase. This was true for the p21^−/−^ MFs as well, suggesting that p21 was not responsible for the observed block in gDNA replication (Fig. S5). We next examined the role of contact inhibition in wild-type MFs and, to our surprise, when we removed contact inhibition from infected wild-type MFs, we observed, for the first time, the restoration of gDNA replication that was also completely dependent on the presence of HCE1/TEEGR. This observation demonstrated that contact inhibition was indeed playing a role in suppressing gDNA replication in infected confluent monolayers. However, this also highlighted that there was an additional cell-intrinsic block in S-phase progression that seemed to be operating in our HFF and FUCCI cells. This result raises the question as to what cell-intrinsic differences are responsible for this block in HFFs and FUCCI NIH 3T3 cells (72). Although still unclear, if we compare our two primary fibroblast cell lines (HFFs and MFs), it is worth noting that our in-house-derived MFs were freshly isolated and at a low passage number (~1 to 3), while our HFFs were often used after 20+ passages. The effects of high passage number and continuous culture may have, unexpectedly, selected for primary fibroblasts that have significantly modified their cell cycle programs and thus are altered in their sensitivity to culture conditions or even infection. This observation does raise the possibility that even primary cell lines can respond differently to infection based on their origin or passage history.

10.1128/msphere.00160-22.4 FIG S4 Toxoplasma-infected cancerous cell lines are not blocked in DNA replication. (A) Cells from various immortalized/cancerous cell lines were either uninfected (in presence of low or high serum) or infected with wild-type or HCE1/TEEGR knockout T. gondii parasites for 20 h in the presence of EdU. Monolayers were fixed after 20 h and stained for EdU incorporation using Alexa Fluor 488 (green). The percentages of EdU-positive nuclei are shown. (B) Graphical representation from the data in panel A. Download FIG S4, TIF file, 1.5 MB. Copyright © 2022 Pierre-Louis et al. 2022 Pierre-Louis et al. https://creativecommons.org/licenses/by/4.0/ This content is distributed under the terms of the Creative Commons Attribution 4.0 International license.

10.1128/msphere.00160-22.5 FIG S5 TgHCE1/TEEGR does not activate DNA replication in mouse fibroblast *Δp21* cells. (A) Mouse fibroblast *Δp21* cells were incubated with EdU and either uninfected with 1% or 20% serum or infected with TgHCE1/TEEGR-Ty (wild-type) in 20% serum. Monolayers were fixed after 24 h and stained for EdU incorporation using Alexa Fluor 488 (green), GAP45 antibody label parasite membrane (red), and DAPI nuclei (blue). Scale, 1,000 μm. Download FIG S5, TIF file, 2.8 MB. Copyright © 2022 Pierre-Louis et al. 2022 Pierre-Louis et al. https://creativecommons.org/licenses/by/4.0/ This content is distributed under the terms of the Creative Commons Attribution 4.0 International license.

It still remains to be determined exactly how T. gondii benefits from driving its infected host cell into the cell cycle since, at least for the tachyzoite form of the parasite, the lytic cycle is extremely rapid. At the moment we cannot rule out the possibility that the main function of HCE1/TEEGR is, in fact, to manipulate the NF-κB response to infection, with the concomitant influence on the host cell cycle being simply a secondary effect. Regardless, the overlap in these pathways is intriguing, as a number of studies have observed significant levels of cross talk between the NF-κB signaling pathway and progression through the cell cycle (73). In considering this parasite’s desire to influence its host cell cycle, it has been shown that T. gondii appears to have an innate preference for host cells that are in the G_1_ or S-phases of the cell cycle while seeming to avoid infecting cells that are in G_2_/M (74). Additionally, T. gondii exhibits a diminished rate of growth and an increased tendency to convert into cyst-forming bradyzoites in host cells that overexpress autoantigen-1, a negative regulator of the cell cycle (75). These phenomena suggest that the parasite benefits in some way by choosing to infect host cells with the capacity to enter into S-phase during the lytic cycle. Although our studies have yet to determine the mechanistic basis for the block in gDNA synthesis in HFF or FUCCI cells, it is likely that these phenomena are anomalous, as recently derived, and arguably more physiologically relevant, host cell fibroblasts can be driven to replicate their genomes through the actions of HCE1/TEEGR. Further studies will be necessary to determine if the duplication of the host genome occurs in other low-passage-number mammalian cell types and if this provides any benefit for parasite expansion or long-term persistence during *in vivo* animal infections.

## MATERIALS AND METHODS

### Parasite and cell culture.

T. gondii tachyzoites were maintained by serial passage in both human telomerase reverse transcriptase (hTERT) and human foreskin fibroblast (HFF) monolayers grown in complete Dulbecco’s modified Eagle’s medium (DMEM) containing 4.5 g/liter glucose, 4 mM l-glutamine, 1× penicillin-streptomycin solution (Corning) with 10% cosmic calf serum (CCS) at 37°C in 5% CO_2_. Parasite strains used in this study are listed in Table S1 in the supplemental material. Primer sets and plasmids are listed in Tables S2 and S3. Stable transgenic parasites were selected in 25 μg/mL mycophenolic acid (MPA) and 25 μg/mL xanthine (Xa), 3 mM pyrimethamine (Pyr), or fluorodeoxyribose resistance (FUDR) (Sigma).

10.1128/msphere.00160-22.6 TABLE S1 **Toxoplasma gondii** parasite strains used in this study. List of major T. gondii strains, their genotypes, and the drug markers used in this study. Download Table S1, DOCX file, 0.02 MB. Copyright © 2022 Pierre-Louis et al. 2022 Pierre-Louis et al. https://creativecommons.org/licenses/by/4.0/ This content is distributed under the terms of the Creative Commons Attribution 4.0 International license.

10.1128/msphere.00160-22.7 TABLE S2 Primers used for cloning of **Toxoplasma gondii** genes, vectors, and PCR validation. List of the major primers used to clone genes, generate vectors, repair templates, and perform diagnostic assays in this study is shown. Download Table S2, DOCX file, 0.03 MB. Copyright © 2022 Pierre-Louis et al. 2022 Pierre-Louis et al. https://creativecommons.org/licenses/by/4.0/ This content is distributed under the terms of the Creative Commons Attribution 4.0 International license.

10.1128/msphere.00160-22.8 TABLE S3 Plasmids used in this study. Download Table S3, DOCX file, 0.03 MB. Copyright © 2022 Pierre-Louis et al. 2022 Pierre-Louis et al. https://creativecommons.org/licenses/by/4.0/ This content is distributed under the terms of the Creative Commons Attribution 4.0 International license.

The CRISPR-Cas9 system was used to generate endogenous tagged, gene-disrupted, and complemented strains (39, 76). For this study, all the CRISPR/Cas9 vectors (pSAG1::Cas9-U6::sgRNA [variable region]) were generated in a fashion similar to that by Shen et al. (39) to change the UPRT targeting gRNA to other specific guide sgRNA listed in Table S2. To generate the endogenous TgRH *Δku80*, *Δhxgprt*, *hce1/teegr-*Ty-tagged line, the TgRH *Δku80*, *Δhxgprt* was used. About 1 kb of *hce1/teegr* gene was amplified from the parental genomic DNA via PCR (Table S2). The resulting fragment, placed in frame with the Ty epitope tag followed by a stop codon, was subsequently inserted via Gibson assembly into the pLIC vector harboring the *hxgprt* gene that can be used as a selection cassette. The targeting vector along with the (pSAG1::Cas9-U6::sgHCE1/TEEGR) construct were used for cotransfection of hTERT cells with parental parasites and selected with the appropriate drugs (Table S1). This CRISPR/Cas9 vector (pSAG1::Cas9-U6::sgHCE1/TEEGR) was generated with *hce1/teegr* targeting sgRNA via PCR mutagenesis in the original CRISPR/Cas9 plasmid (pSAG1::Cas9-U6::sgUPRT). The Q5 site-directed mutagenesis kit (New England BioLabs) was used to perform the PCR mutagenesis with pSAG1::Cas9-U6::sgUPRT plasmid as the template. Parasites were single-cloned into 96-well plates by limiting dilution and screened for endogenous integration at the correct locus via IFA (immunofluorescence imaging) and PCR (Fig. 1C and Table S2 and Fig. S1C).

To generate the HCE1/TEEGR knockout line TgRH *Δku80*, *Δhxgprt*, *hce1/teegr-*Ty-HXGPRT, *Δhce1/teegr-*Ty::Pyr, the endogenous tagged line TgRH *Δku80*, *Δhxgprt*, *hce1/teegr-*Ty-HXGPRT was used. First, the pSAG1::Cas9-U6::sgUPRT vector was altered to sgRNA targeting the *hce1/teegr* gene to generate the CRISPR cutting vector pSAG1::Cas9-U6::sg*hce1/teegr* cutting using Q5 site-directed mutagenesis kit (New England BioLabs). Second, the DHFR drug marker amplicon with 40-bp homology flanking region to *hce1/teegr* was amplified. The resulting CRISPR cutting vector, pSAG1::Cas9-U6::sg*hce1/teegr* cutting, was used along with the DHFR amplicon to subsequently transfect the endogenous HCE1/TEEGR-Ty-tagged parasites TgRH *Δku80*, *Δhxgprt*, *hce1/teegr-*Ty*-HXGPRT*. The parasites were then selected with pyrimethamine (Table S1) and were screened via IFA and PCR for gene disruption of the *hce1/teegr* gene (Fig. 1C and Table S2 and Fig. S1C).

The complemented strain TgRH *Δku80*, *Δhxgprt*, *hce1/teegr-*Ty-HXGPRT, *Δhce1/teegr-*Ty::Pyr, *Δuprt*::*hce1/teegr*-HA-Fudr was generated using the pUPRT-*vha1* cDNA-3xHA shuttle vector (77), a gift from Moreno Silvia, University of Georgia Athens, which contains the 5′ and 3′ untranslated regions (UTRs) of the *uprt* gene (78, 79) and the corresponding CRISPR vector. A generated pUPRT-*hce1/teegr*-HA vector was first assembled via Gibson assembly (NEB) using amplicon from the pUPRT-*vha1* cDNA-3×HA vector and the amplicon from genomic DNA wild-type RH *Δku80*, *Δhxgprt* for *hce1/teegr*. The CRISPR plasmid was then generated as described above with the corresponding sgRNA for the *uprt* gene locus to construct pSAG1::Cas9-U6::sgUPRT. Both plasmids were subsequently used to cotransfect knockout parasites TgRH *Δku80*, *Δhxgprt*, *hce1/teegr-*Ty-HXGPRT, *Δhce1/teegr-*Ty::Pyr and then selected for FUDR resistance to successfully generate the complemented strain TgRH *Δku80*, *Δhxgprt*, *hce1/teegr-*Ty-HXGPRT, Δ*hce1/teegr-*Ty::Pyr, Δ*uprt*::*hce1/teegr*-HA-Fudr via limiting dilution. Complementation was further assessed by visualizing the effector translocation via IFA and PCR for gene insertion and crossover was checked for the *hce1/teegr* and *uprt* (Fig. 1C and Table S2 and Fig. S1C). Additional primers were used to check each strain lysate for positive ROP18 Toxoplasma gondii and construct the pUltra/eGFP-HCE1/TEEGR-Ty mammalian expression vector.

### Immunofluorescence assays.

HFF cells were grown on 12-mm glass coverslips to confluence and then infected. Samples were fixed with 4% formaldehyde in phosphate-buffered saline (PBS) for 10 min and then permeabilized with 0.5% Triton X-100 in PBS for 10 min and blocked in 5% CCS in PBS for 30 min. Cells were incubated with primary antibodies for 30 to 60 min and then washed three times in PBS (see Table S4 for primary antibodies used). Secondary antibodies, goat anti-mouse IgG Alexa Fluor 488 and goat anti-rabbit IgG Alexa Fluor 594 (Life Technologies), as well as DAPI, were added for 30 to 60 min, followed by a PBS wash. Coverslips were mounted with Fluoro-Gel (Electron Microscopy Sciences), and samples were examined using a Lionheart FX automated microscope (BioTek Instruments, Inc.).

10.1128/msphere.00160-22.9 TABLE S4 Antibodies and reagents used in this study. List of the various antibodies used in immunofluorescence assays (IFA) and Western blot analyses as well as specific reagents used in this study is shown. Download Table S4, DOCX file, 0.03 MB. Copyright © 2022 Pierre-Louis et al. 2022 Pierre-Louis et al. https://creativecommons.org/licenses/by/4.0/ This content is distributed under the terms of the Creative Commons Attribution 4.0 International license.

### Plaque assay.

Confluent monolayers of HFF cells grown in 6-well plates were infected with 100 tachyzoites/well. Plates were incubated at 37°C with 5% CO_2_ for 7 days without disruption. On day 7, the wells were washed once with PBS and incubated in 100% ethanol for 5 min, followed by staining with crystal violet (CV). Plates were washed with deionized (DI) water, air dried, and visualized using the ChemiDocTM MP Imaging System (Bio-Rad).

### RNA-Seq mapping and differential expression analysis.

Six samples, corresponding to three biological replicates each for WT and KO, had their paired-end short-read RNA-Seq sequences individually aligned to the human reference genome sequence (GRCh38/hg38) using the software HISAT2 (PMID 31375807) under default parameters. For the differential expression analysis, we used HTseq v.2.1.0 (PMID 25260700) and Bioconductor/DESEq2 (PMID 25516281). HTseq-count tool was used to transform genetic depth information into a count of readings by gene overlapping into the gtf annotation of GRCh38 genome. Count output files were obtained for each replicate for each condition (WTs and KOs). The DESeq2 Bioconductor R package version 1.26.0 was used to determine differentially expressed genes, data normalization was performed using the median of ratios method, and the default parameters were followed. The transcripts showing a log_2_ fold change of >1.5 and that presented a statistically significant differential expression (adjusted *P* value of <0.05) were selected. The genes differentially expressed by DESeq2 were classified between downregulated and upregulated. Visualization of heatmaps and volcano plots were made by using gplots/heatmap3 and Bioconductor EnhancedVolcano R packages.

### EdU assay.

For flow cytometry, infected and control monolayers were cultured in DMEM complete medium containing 2 μm EdU (5-ethynyl-2′-deoxyuridine) for 24 h. Immunofluorescence assays were performed on HFF cells using the Click-iT EdU Alexa Fluor 488 kit (Invitrogen) and imaged with a Lionheart FX automated microscope (BioTek Instruments, Inc.). FUCCI cells were stained using the Click-iT EdU Alexa Fluor 405 kit (Invitrogen) and analyzed with the CytoFLEX flow cytometer (Beckman Coulter) and FlowJo software (Tree Star).

To assess DNA replication in monolayers, confluent and quiescent HFF cells were infected with wild-type or mutant parasites for about 4 to 6 h, and positive-control monolayers were cultured in DMEM complete medium. All monolayers were washed with HBSS, trypsinized, and resuspended with either 1 or 20% CCS DMEM depending on the condition of the experiment; the cells were subsequently seeded to new coverslips, with 60% to 80% confluence per well. Analysis of the percentage of cells in S-phase was determined by dividing the number of EdU incorporation in actively growing cells (Alexa Fluor 488) by the total number of cells labeled with DAPI nuclei. GAP45 antibody was used to stain parasite membrane.

### Wound healing assay in MF cells.

Mice ears were obtained from the C57BL/6 mouse wild type for tissue biopsy by following the Jaenisch Lab protocol by Kathy Hoover of the Jones Lab, with a few modifications done by Tarleton lab at University of Georgia Athens (UGA) and our group. The ears were gifts from the Kim Klonowsky lab at UGA, and the mice were purchased from Charles River Laboratories (Wilmington, MA). These cells were harvested and at confluence were passed or frozen for future experiments. They were then cultured in DMEM complete medium containing 10% CCS or fetal bovine serum (FBS). All monolayers were washed with HBSS (Hanks’ balanced salt solution), trypsinized, and resuspended with either 10% CCS or FBS, and then the cells were subsequently seeded to culture insert 4-well Ibidi u-Dish^35mm^ at 60% to 80% confluence per well in a 5% CO_2_ high-humidity incubator at 37°C. The cells were infected with the appropriate parasite lines for about 4 h, resulting in a complete monolayer invasion, and then the monolayer was gently washed with 1% CCS and subsequently cultured with 2 μM EdU for about 18 to 20 h for the MF cells and 24 h for the HFF and FUCCI cells before the wound get completely saturated with cells, if any. Immunofluorescence and Click-iT EdU assays were subsequently performed on the MF (or HFF and FUCCI cells) to analyze cells in S-phase as described above.

### Western blotting.

HFF cells were collected at 8, 16, and 24 h postinfection. Monolayers were lysed in 100 μL of 1× Laemmli buffer, resolved by SDS-PAGE, and transferred onto nitrocellulose membranes. Immunoblots were probed with primary antibodies in 3% milk in PBS for 1 h (see Table S4 for the antibodies used), washed in 0.5% PBS–Tween, and then incubated for 1 h with secondary antibodies conjugated to IRDye 680CW (goat anti-rabbit or goat anti-mouse) or IRDye 800CW (goat anti-rabbit or goat anti-mouse) (LI-COR Biosciences) and signals detected using the ChemiDoc MP imaging system (Bio-Rad).

### FUCCI cell cycle analysis.

FUCCI cells were seeded in 24-well plates and grown to confluence. Cells were synchronized to G_1_ phase (red) by serum depletion 24 h prior to infection. Parasites were collected and washed once in HBSS. Monolayers were infected at an MOI of 20. Using the Lionheart FX automated microscope (BioTek Instruments, Inc.), the plate was incubated at 37°C with 5% CO_2_, and time course images were collected every 10 min for 20 h. Red and green FUCCI cells were counted for each time point using Gen5 3.0 software (BioTek Instruments, Inc.).

### Phylogenetic analysis.

Orthologs of TGGT1_239010 from reference strains of 12 different haplogroups of T. gondii and an out group of Hammondia hammondi were retrieved from ToxoDB (www.toxodb.org) and aligned using Clustal (80) with a gap opening penalty of 30 and extension penalty of 0.75. The phylogeny was inferred by using a maximum likelihood method and JTT protein-based substitution model (81) in MEGA X (82) based on 1,000 bootstrap replicates (83). Initial tree(s) for the heuristic search was obtained automatically by applying the maximum parsimony method.

### Data availability.

All RNA-Seq data generated in this study are available in the NCBI sequence read archive (SRA) under the BioProject no. PRJNA828190.
