# Supplementary figures and images for: Disruption of Toxoplasma gondii-Induced Host Cell DNA Replication Is Dependent on Contact Inhibition and Host Cell Type
Source: mSphere. 2022 May 19;7(3):e00160-22. doi: 10.1128/msphere.00160-22 (PMC9241542; doi:10.1128/msphere.00160-22)

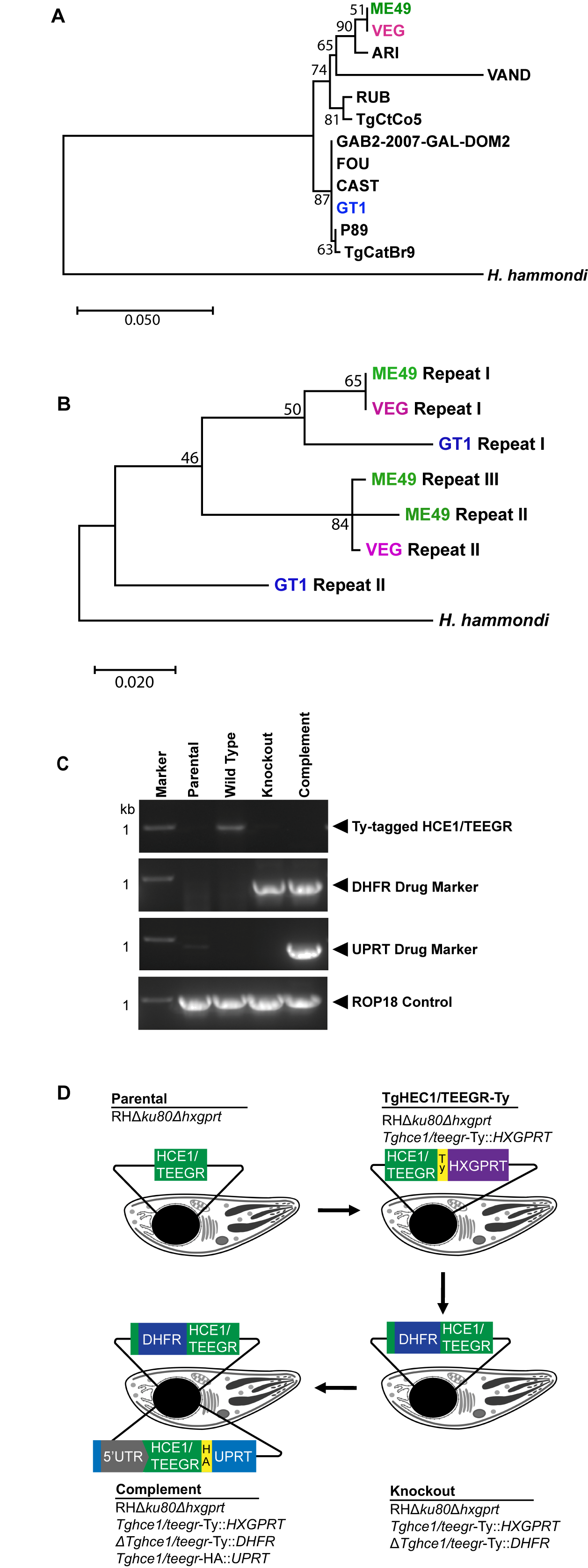

Supplement: FIG S1 [file msphere.00160-22-s0001.tif]

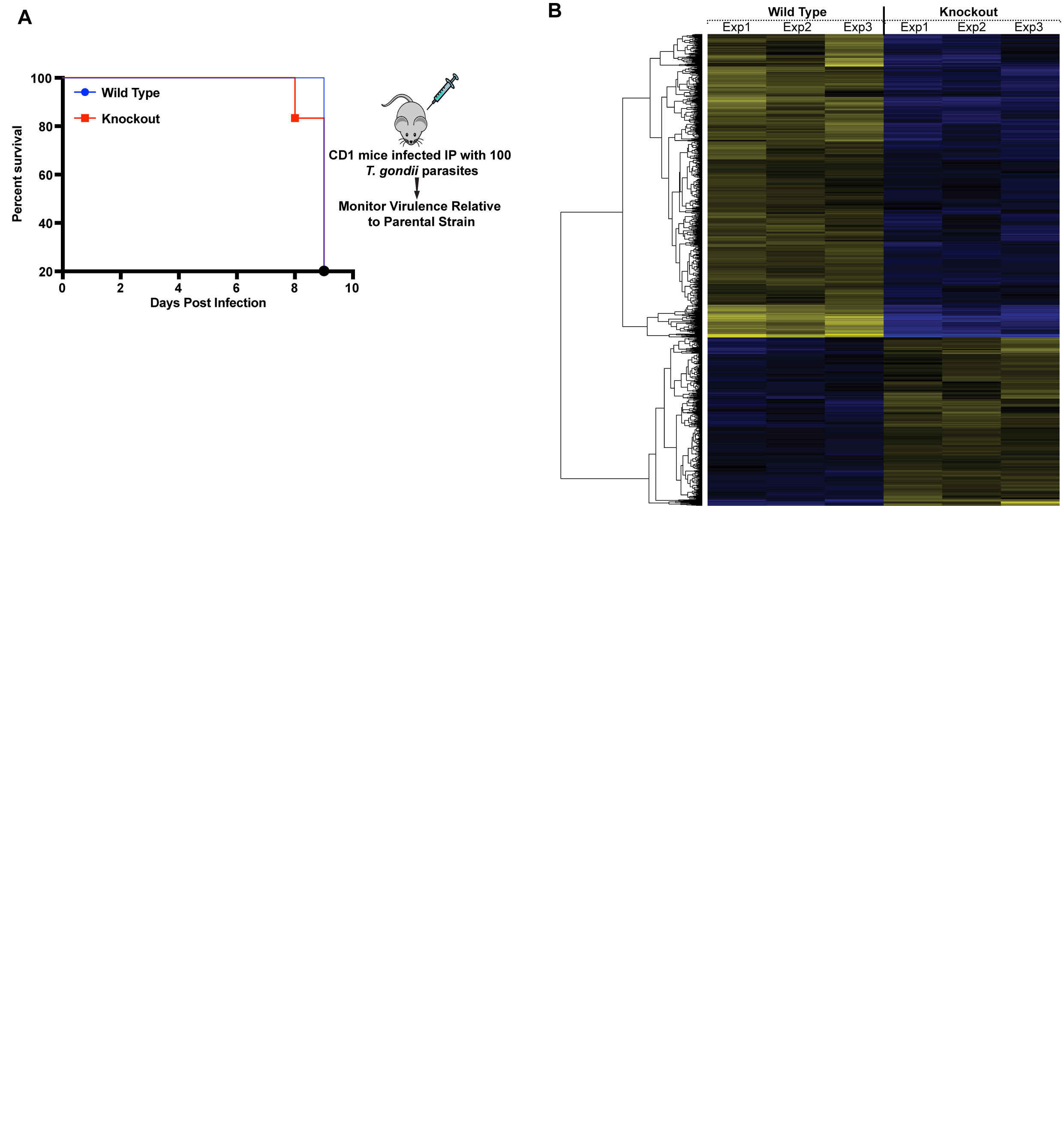

Supplement: FIG S2 [file msphere.00160-22-s0002.tif]

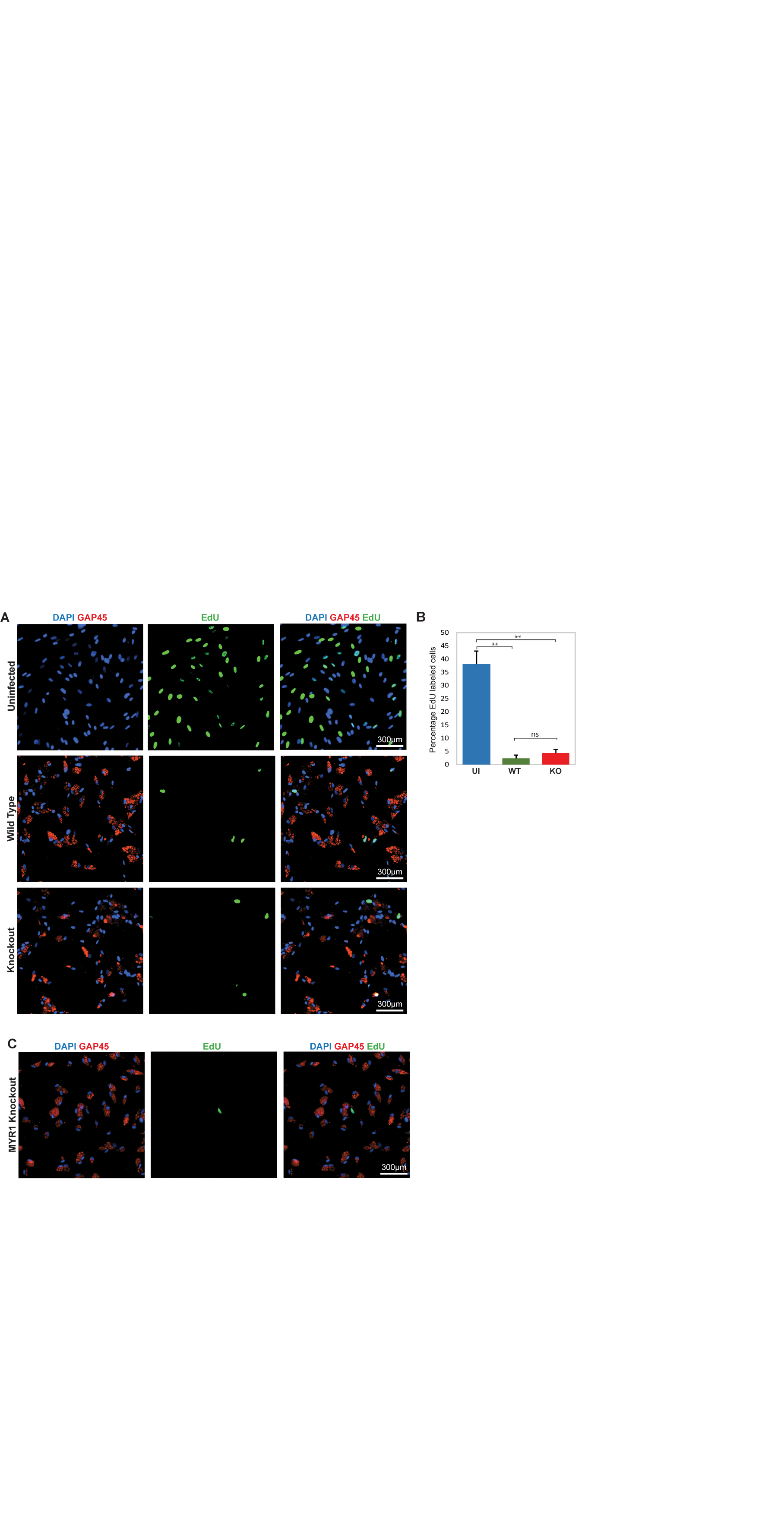

Supplement: FIG S3 [file msphere.00160-22-s0003.tif]

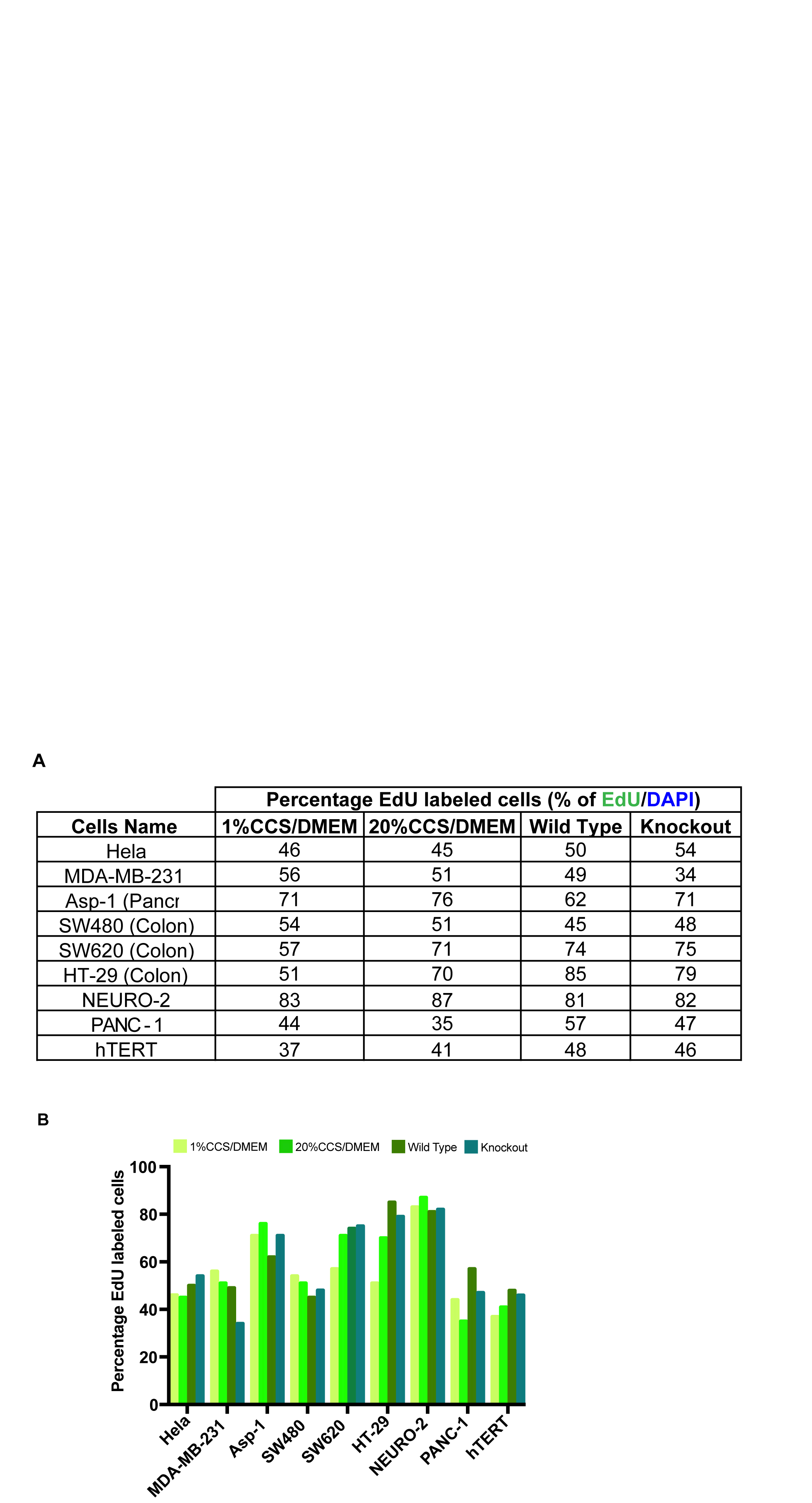

Supplement: FIG S4 [file msphere.00160-22-s0004.tif]

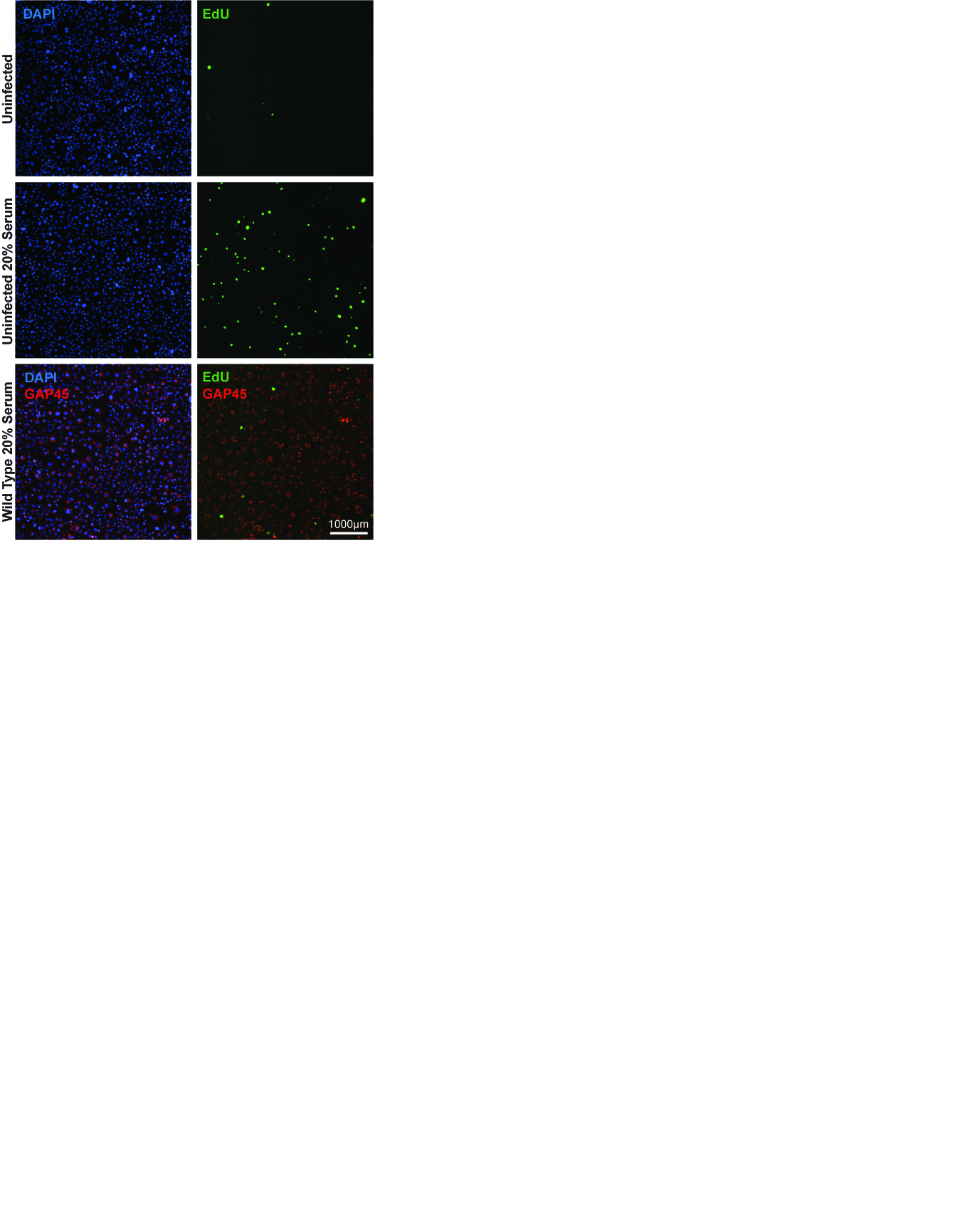

Supplement: FIG S5 [file msphere.00160-22-s0005.tif]
