# Supplementary material for: Disruption of Toxoplasma gondii-Induced Host Cell DNA Replication Is Dependent on Contact Inhibition and Host Cell Type
Source: mSphere. 2022 May 19;7(3):e00160-22. doi: 10.1128/msphere.00160-22 (PMC9241542; doi:10.1128/msphere.00160-22)
Supplement: TABLE S1 [file msphere.00160-22-s0006.docx]

Table S1. *Toxoplasma gondii* parasite strains used in this study.

| **Common Name** | **Genotype** | **Drug Selection** | **Description** |
| --- | --- | --- | --- |
| RH *Δku80 Δhxgprt* (Parental) | TgRH *Δku80, Δhxgprt* | N/A | Parental type I RH *T. gondii* used as background strain to make mutant lines. |
| RH *Δku80 Δhxgprt* TgHCE1/TEEGR-Ty  (TgHCE1/TEEGR-Ty) | TgRH *Δku80, Δhxgprt,* *hce1/teegr-*Ty*-*HXGPRT | MPA/Xan | Tg*hce1/teegr* endogenously-tagged line with insertion of HXGPRT drug selection marker in Tg*hce1/teegr* coding region. |
| RH *Δku80 Δhxgprt* TgΔHCE1/TEEGR-Ty *(*Knockout or TgΔHCE1/TEEGR-Ty) | TgRH *Δku80, Δhxgprt,* *hce1/teegr-*Ty*-*HXGPRT, Δ*hce1/teegr-*Ty::Pyr, | Pyr | Tg*hce1/teegr* disruptant mutant line with insertion of DHFR drug selection marker in Tg*hce1/teegr* coding region and integrants were selected for pyrimethamine resistance. |
| RH *Δku80 Δhxgprt* TgHCE1/TEEGR-HA (Complement) | TgRH *Δku80, Δhxgprt,* *hce1/teegr-*Ty*-*HXGPRT, *Δhce1/teegr-*Ty::Pyr, *Δuprt*::*hce1/teegr*-HA-Fudr | FUDR | The RH Tg*hce1/teegr* Knockout line is used where HA-tagged Tg*hce1/teegr* was inserted into the UPRT locus and integrants were selected for FUDR resistance. |
