# Supplementary material for: Disruption of Toxoplasma gondii-Induced Host Cell DNA Replication Is Dependent on Contact Inhibition and Host Cell Type
Source: mSphere. 2022 May 19;7(3):e00160-22. doi: 10.1128/msphere.00160-22 (PMC9241542; doi:10.1128/msphere.00160-22)
Supplement: TABLE S2 [file msphere.00160-22-s0007.docx]

Table S2. Primers used for cloning of *Toxoplasma* *gondii* genes, vectors and PCR validation.

| **Number**  **& Name** | **#** | **Sequence (5’ to 3’)** | **Polarity** |
| --- | --- | --- | --- |
| Fw for *hce1/teegr* gene ~999kb fragment I05 | 1 | AACCTCAGCCTTTCGCTGTACGCCTACAGACTCCGCTAGA | sense |
| Rev for *hce1/teegr* gene ~999kb fragment I04 | 2 | GAATGTCGGACGGATCTTCCGAGGTCCACACGAACCAGGA | anti-sense |
| Fw for pLIC *hce1/teegr*-Ty tagging I03 | 3 | GAATGTCGGACGGATCTTCCGAGGTCCACACGAACCAGGA | sense |
| Rv for pLIC *hce1/teegr*-Ty tagging I06 | 4 | AACCTCAGCCTTTCGCTGTACGCCTACAGACTCCGCTAGA | anti-sense |
| Fw with sgRNA for HCE1/TEEGR-Ty Tagging H99 | 5 | CGGATCTTCCTGACATCAGTGTTTTAGAGCTAGAAATAGC | sense |
| Rev for CRISPR HCE1/TEEGR-Ty tagging F4 | 6 | GTAAATGGGGATGTCAAGTT | anti-sense |
| A33 PCR Diagnostic Fw | 7 | TGCCACAGCGGCTCCCCCACAGATT | sense |
| A34 PCR Diagnostic Rev | 8 | GTCCAGGGGGTCCTGGTTGGTGTGCACCTC | anti-sense |
| K80 PCR Diagnostic Fw | 9 | CAGTCAAAGCAAGCGCAATTTGCACCAAGCCC | sense |
| K81 PCR Diagnostic Rev | 10 | GCGGTATCGGCTCTCCCAGTGGTGGCACAAATG | anti-sense |
| Fw sgRNA/hr (for *hce1/teegr* cutting (KO)) | 11 | GTTCCTTTACAGACTCCCCGTTTTAGAGCTAGAAATAGC | sense |
| DHFR fw with 40bd HR | 12 | AACCTCAGCCTTTCGCTGTAATGAGGACCGGCGATCACCGCACAGGGGTGCGACTCATCA | sense |
| DHFR Rev with 40bd HR | 13 | TTCGAGCCGATCCCGAAGAAGAATGTCGGACGGATCTTCCTAGAATTCATGGTGAGCAAG | anti-sense |
| PCR Diagnostic Fw 5’UTR + I12 | 14 | GCATTCATGTGAAGAGCTGGCGGTGTG | sense |
| PCR Diagnostic Rev 5’UTR +A65 | 15 | GTCGAACAAAGCACGGAGGAGAGACGGAAAG | Anti-sense |
| A64 (fw 3’UPRT) | 16 | ACGACGTCCCGGACTACGCTTAAGCTAGCGTCTCTAGTTTTTTTGACAGACCGCTGACGG | sense |
| A15 (Rev UPRT) | 17 | GACGGTTCACTCACTTCACGTTTAGAAGCCCTGTGGACAGGTCCGACGAA | anti-sense |
| A18 (fw *hce1/teegr* gene) | 18 | GGCTTCTAAACGTGAAGTGAGTGAACCGTCGGTTCTTGAGAAAACAGGCA | sense |
| A63 (rev *hce1*/teegrgene+HA end) | 19 | CGAAGAAGAATGTCGGACGGATCTTCCGGTACCTACCCGTACGACGTCCCGGACTACGCT | Anti-sense |
| sgRNA for UPRT A19 | 20 | TCTAGACTTTCAACTGACGTGTTTTAGAGCTAGAAATAGC | Sense |
| A35 PCR Diagnostic Fw | 21 | GACGTGGGAGCAGGTGACGGATTACTCGCC | sense |
| A36 PCR Diagnostic Rev | 22 | CGCCTCGTAAACATTCCCGTTACAGGTGTA | Anti-sense |
| C88 PCR Diagnostic Fw for ROP18 | 23 | GCGACAGAAAGCACTCGAGACGTTTCATTG | Sense |
| C89 PCR Diagnostic Rev for ROP18 | 24 | CTTTCAAGAGGAGGAAATTCGCCGGTTTG | Anti-sense |
| A90 HCE1/TEEGR-Ty Fw pultra | 25 | GCATGGACGAGCTGTACAAGATGACGCCATGGTTGGCTTTTGCAAGCGCC | sense |
| B10 HCE1/TEEGR-Ty Rev pUltra | 26 | GAGGTGCACACCAACCAGGACCCCCTGGACTGATCAGAATTCGTTCCGGA | Anti-sense |
| A97 pUltra fw | 27 | TGATCAGAATTCGTTCCGGAGTCGTCGACTCGACAATCAA | Sense |
| B6 pUltra rev | 28 | CCGGGATCACTCTCGGCATGGACGAGCTGTACAAG | Anti-sense |
| M83 Seq primer | 29 | GGTGGCTCACTGGTTCATCTACAGGAAGC | sense |
| T3 for CRISPR  Seq + | 30 | TCCCTTTAGTGAGGGTTAAT | Anti-sense |
| EGFP_C_Primer | 31 | CATGGTCCTGCTGGAGTTCGTG | sense |
|  |  |  |  |
