# Supplementary material for: Disruption of Toxoplasma gondii-Induced Host Cell DNA Replication Is Dependent on Contact Inhibition and Host Cell Type
Source: mSphere. 2022 May 19;7(3):e00160-22. doi: 10.1128/msphere.00160-22 (PMC9241542; doi:10.1128/msphere.00160-22)
Supplement: TABLE S3 [file msphere.00160-22-s0008.docx]

Table S3. Plasmids used in this study

|   **Common Name**   | **Description** |   **Source**   |
| --- | --- | --- |
| pSAG1-CAS9-U6gRNA (UPRT) | Vector expression a fused CRISPR/Cas9 to GFP with a gRNA target sequence within the UPRT locus driven by the U6 promoter. It was then used in mutagenesis to create HCE1/TEEGR and UPRT targeted CRISPR cutting vectors. | This paper |
| pLIC-YFP-HXGPRT   | Vector containing a ligation independent cloning site (LIC) fused in frame to YFP and the HXGPRT *T. gondii* selectable drug marker. | From Vernon B. Carruthers’ lab   |
|   pUltra/eGFP Mammalian Expression Vector | A human ubiquitin C (hUbC) promoter driven vector used to clone in Tg*hce1/teegr*- EGFP for expression in transient transfection in HFF cells. |   Gift from Silvia Moreno’s Lab |
|  |  |  |
