# Supplementary material for: Disruption of Toxoplasma gondii-Induced Host Cell DNA Replication Is Dependent on Contact Inhibition and Host Cell Type
Source: mSphere. 2022 May 19;7(3):e00160-22. doi: 10.1128/msphere.00160-22 (PMC9241542; doi:10.1128/msphere.00160-22)
Supplement: TABLE S4 [file msphere.00160-22-s0009.docx]

Table S4. Antibodies and reagents used in this study.

| **Category** | **Target (clone)** | **Species** | **Source** | **Dilution** | **Product #** |
| --- | --- | --- | --- | --- | --- |
| Immunofluorescence Assays: Primary | Ty-Tag | mouse | In house | 1/1000 |  |
|  | GAP45 | rabbit | In house/Lampire Biologicals | 1/1000 |  |
|  | GRA7 | rabbit | David Sibley | 1/1000 |  |
|  | Cyclin E (E-4) | mouse | Santa Cruz | 1/1000 | sc-377100 |
|  | HA-Tag (6E2) | mouse | Cell Signaling | 1/1000 | 2367 |
|  | GFP | rabbit | Invitrogen | 1/200 | A6455 |
| Immunofluorescence Assays: Secondary | Mouse IgG | Goat conjugated to Alexa488 | Life Technologies | 1/1000 | A11029 |
|  | Rabbit IgG | Goat conjugated to Alexa594 | Life Technologies | 1/1000 | A11037 |
| Western Blotting: Primary | Ty-Tag | mouse | In house | 1/1000 |  |
|  | GAP45 | rabbit | In house | 1/1000 |  |
|  | HA-Tag (6E2) | mouse | Cell Signaling | 1/1000 | 2367 |
|  | GFP | rabbit | Invitrogen | 1/2000 | A6455 |
|  | Cyclin E (HE12) | mouse | Santa Cruz | 1/1000 | sc-247 |
|  | hFAB^TM^ Rhodamine Anti-Actin | human | Bio-Rad | 1/1000 | 12004163 |
| Western Blotting: Secondary | Mouse IgG | Goat conjugated to IRDye 680CW | LI-COR Biosciences | 1/2000 | 926-68070  lot#: C80619-05 |
|  | Mouse IgG | Goat conjugated to IRDye 800CW | LI-COR Biosciences | 1/1000 | 926-32210  lot#: C60726-02 |
| Betadine Solution |  |  | Fisher Scientific |  | 19027132 |
| Liberase TM |  |  | Roche |  | 05401020001 |
| Penicillin Streptomycin |  |  | Cytiva / Hyclone |  | SV30010 |
| Hanks’ Balanced Salt Solution |  |  | Corning |  | 55-022-PB  Lot#: 31420016 |
